# Deciphering MCR-2 Colistin Resistance

**DOI:** 10.1128/mBio.00625-17

**Published:** 2017-05-09

**Authors:** Jian Sun, Yongchang Xu, Rongsui Gao, Jingxia Lin, Wenhui Wei, Swaminath Srinivas, Defeng Li, Run-Shi Yang, Xing-Ping Li, Xiao-Ping Liao, Ya-Hong Liu, Youjun Feng

**Affiliations:** a Department of Medical Microbiology and Parasitology, Zhejiang University School of Medicine, Hangzhou, Zhejiang, China; b National Risk Assessment Laboratory for Antimicrobial Resistance of Animal Original Bacteria, South China Agricultural University, Guangzhou, Guangdong, China; c Department of Biochemistry, University of Illinois, Urbana, Illinois, USA; d Institute of Biophysics, Chinese Academy of Sciences, Beijing, China; University of British Columbia

**Keywords:** colistin resistance, dissemination, domain swapping, MCR-1, MCR-2, origin, plasmid transfer, structure-guided mutagenesis

## Abstract

Antibiotic resistance is a prevalent problem in public health worldwide. In general, the carbapenem β-lactam antibiotics are considered a final resort against lethal infections by multidrug-resistant bacteria. Colistin is a cationic polypeptide antibiotic and acts as the last line of defense for treatment of carbapenem-resistant bacteria. Very recently, a new plasmid-borne colistin resistance gene, *mcr-2*, was revealed soon after the discovery of the paradigm gene *mcr-1*, which has disseminated globally. However, the molecular mechanisms for MCR-2 colistin resistance are poorly understood. Here we show a unique transposon unit that facilitates the acquisition and transfer of *mcr-2*. Evolutionary analyses suggested that both MCR-2 and MCR-1 might be traced to their cousin phosphoethanolamine (PEA) lipid A transferase from a known polymyxin producer, *Paenibacillus*. Transcriptional analyses showed that the level of *mcr-2* transcripts is relatively higher than that of *mcr-1*. Genetic deletions revealed that the transmembrane regions (TM1 and TM2) of both MCR-1 and MCR-2 are critical for their location and function in bacterial periplasm, and domain swapping indicated that the TM2 is more efficient than TM1. Matrix-assisted laser desorption ionization–time of flight mass spectrometry (MALDI-TOF MS) confirmed that all four MCR proteins (MCR-1, MCR-2, and two chimeric versions [TM1-MCR-2 and TM2-MCR-1]) can catalyze chemical modification of lipid A moiety anchored on lipopolysaccharide (LPS) with the addition of phosphoethanolamine to the phosphate group at the 4′ position of the sugar. Structure-guided site-directed mutagenesis defined an essential 6-residue-requiring zinc-binding/catalytic motif for MCR-2 colistin resistance. The results further our mechanistic understanding of transferable colistin resistance, providing clues to improve clinical therapeutics targeting severe infections by MCR-2-containing pathogens.

## IMPORTANCE

Carbapenem and colistin are the last line of refuge in fighting multidrug-resistant Gram-negative pathogens. MCR-2 is a newly emerging variant of the mobilized colistin resistance protein MCR-1, posing a potential challenge to public health. Here we report transfer of the *mcr-2* gene by a unique transposal event and its possible origin. Distribution of MCR-2 in bacterial periplasm is proposed to be a prerequisite for its role in the context of biochemistry and the colistin resistance. We also define the genetic requirement of a zinc-binding/catalytic motif for MCR-2 colistin resistance. This represents a glimpse of transferable colistin resistance by MCR-2.

## INTRODUCTION

Antibiotic resistance has been developing as a great challenge to global public health. Global infection by multidrug-resistant (MDR) pathogens is anticipated to cause more than 700,000 deaths (with 214,000 neonatal sepsis deaths) each year (1, 2). As a type of β-lactam antibiotic, carbapenems are generally used as the last resort against lethal infections by multidrug-resistant Gram-negative pathogens (3). Unfortunately the emergence of the notorious New Delhi β-lactamases (NDM-1 [3] and its variants NDM-5 [4], NDM-7 [5], and NDM-9 [6]) in diversified species of *Enterobacteriaceae* (e.g., *Escherichia coli* and *Klebsiella pneumoniae*) has almost pushed us to the cusp of the postantibiotic era. To conquer the carbapenemase (and/or extended-spectrum β-lactamase [ESBL])-producing pathogens, the cationic polypeptide antibiotic colistin (also referred to as polymyxin E; see Fig. S1 in the supplemental material) has to be reintroduced clinically as a choice of priority (7, 8), even though it has an appreciable level of nephrotoxicity and neurotoxicity (9). Given the fact that colistin is used as a veterinary medicine (the old generation of antibiotics) and has been extensively applied in pig and poultry production for over 50 years, it is understood that in sporadic cases, chromosome-encoded colistin resistance is detected, which is frequently associated with point mutations in certain genes: two sets of two-component systems (*pmrAB* in *Salmonella* [10] and *phoPQ* in *K. pneumoniae* [11]) and a regulator gene, *mgrB*, in *K. pneumoniae* (11).

10.1128/mBio.00625-17.1 FIG S1  Chemical structures of the representative polymyxin. (A) Chemical structure of polymyxin E (colistin). (B) Chemical structure of polymyxin B. The letters in red denote positive charge. Download FIG S1, TIF file, 2.5 MB. Copyright © 2017 Sun et al. 2017 Sun et al. This content is distributed under the terms of the Creative Commons Attribution 4.0 International license.

Earlier in 2016, Liu and colleagues (12) reported a new type of transferable colistin resistance, which is not spread by colonial expansion but by plasmid-mediated transmission in *Enterobacteriaceae*. The expression of the mobilized colistin resistance gene *mcr-1* alone conferred the phenotype of robust resistance to colistin (13); the MCR-1 protein product is annotated as a putative member of the phosphoethanolamine (PEA) transferase family. Since its first discovery in southern China in late 2015, the *mcr-1* gene has been found to have disseminated into over 30 countries (with even the mostly developed country, the United States, not exempt [14, 15]) across 5 of 7 continents worldwide (16, 17). This kind of colistin resistance was found to lie in the chemical addition of phosphoethanolamine to the 4′ position of the lipid A moiety of lipopolysaccharide (LPS) on the outer leaflet of the bacterial outer membrane, which consequently gives a significant reduction in the affinity of binding to colistin (11, 17, 18).

Very recently, a novel colistin resistance gene, *mcr-2*, was elucidated by Xavier et al. (19), which exhibits about 76.7% nucleotide (81% amino acid) identity to the paradigm gene, *mcr-1*. In light of the higher prevalence of *mcr-2* in Belgium relative to *mcr-1*, introduction of surveillance of *mcr-2* worldwide is immediately required. In principle, *mcr-2* represents one more threat to public health which parallels that of the *mcr-1* gene. However, we are not aware of the transfer, origin, and mechanism for MCR-2 colistin resistance thus far. Here we report that this is the case through integrative approaches ranging from bioinformatics, comparative genomics, bacterial genetics, structural biology, and molecular microbiology to phylogeny. Certainly, our findings might add new knowledge and close the missing gap in the field of colistin resistance and also be helpful for better monitoring (and control) of the epidemiological dissemination of MCR-2-mediated colistin resistance.

## RESULTS

### Contrasting transmission patterns of MCR-1 and MCR-2.

**FIG 1:**
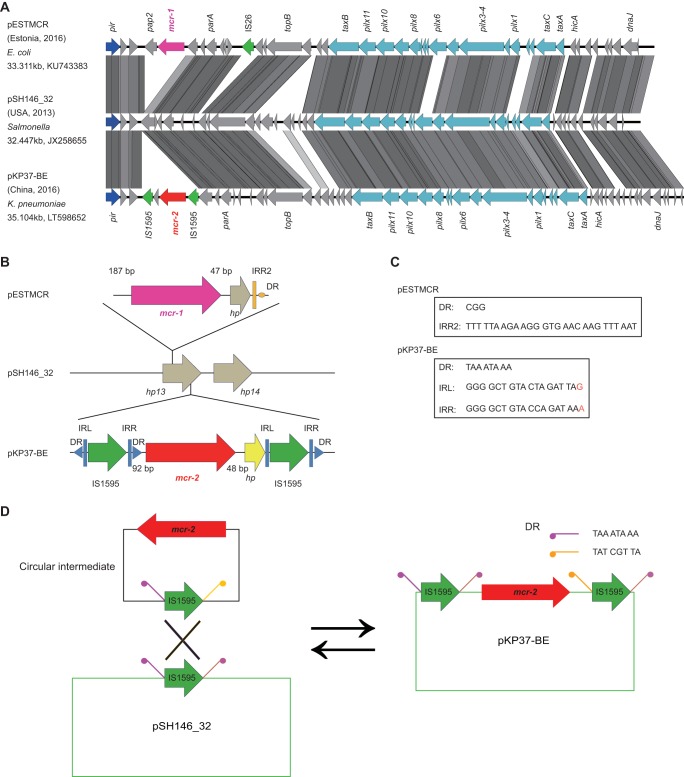
Genetic characterization for the transfer of the *mcr-2* colistin resistance gene. (A) Linear genome comparison of the *mcr-1*/*mcr-2*-harbouring plasmids suggests a molecular mechanism for acquisition of the transfer and acquisition of the *mcr-2*-containing cassette. The genomes used for linear comparison are from the following three plasmids: pESTMCR (GenBank accession no. KU743383), pSH146_32 (GenBank accession no. JX258655), and pKP37-BE (GenBank accession no. LT598652). Boxed arrows represent the position and transcriptional direction of open reading frames (ORFs). Regions of >99% identity are shaded in gray. Genes associated with the *tra*, *pil*, and *vir* loci are colored in light blue, replication-associated genes are shown in dark blue, antibiotic resistance genes are highlighted in red, insertion sequences are indicated in green, and other genes are colored gray. (B) Scheme for the genetic dissection of the *mcr-1*/*mcr-2*-containing cassettes. Resistance genes are indicated with red arrows, while accessory genes are shown with gray arrows. Insertion sequences are highlighted with green arrows labeled with their name or number. The short black arrow denotes the 176-bp spacer between the two insertion events. The yellow vertical black bars represent the transposon inverted region (IR) of IS*Apl1*, while blue vertical black bars correspond to the transposon IR of IS*1595-*like. Direct repeats (DRs) are illustrated by the solid triangle. (C) Genetic feature of the repeat regions. (D) A working model for the insertion and excision of the circularized intermediate of the *mcr-2* antibiotic cassette. Abbreviations: IS, insertion sequence; DR, direct repeat; IRL, inverted region left; IRR, inverted region right; IRR2, the alternate IRR.

Unlike the IncI2-type plasmid pHNSHP45, which is the first identified plasmid carrying the *mcr-1* colistin resistance gene (GenBank accession no. KP347127), the IncX4 family plasmid might represent the most prevalent version of the *mcr-1*-bearing plasmid reservoirs (20, 21). As a novel variant of the *mcr-1* gene, the *mcr-2* gene was first reported in Belgium by Xavier and colleagues in July 2016 (19). Notably, it seems likely that the plasmid pKP37-BE (GenBank accession no. LT598652.1) from porcine and bovine *E. coli* is the only known *mcr-2-*producing plasmid thus far and also can be assigned to the IncX4 replicon type (19). The different adjacent sequences of the *mcr-2* gene from those of *mcr-1* suggested the possibility of varied patterns/modes for the integration, acquisition, or dissemination of the two colistin resistance genes (Fig. 1). To address this hypothesis, using blastn, we performed comparative genomics of the *mcr-2*-positive plasmid pKP37-BE following a search for all of the IncX4 plasmids deposited in the GenBank database (Fig. 1A). The result showed us the similarity of the *mcr-2*-positive plasmid pKP37-BE to *mcr-1*-carrying plasmid pESTMCR (GenBank accession no. KU743383) and highlighted that the IncX4 plasmid pSH146_32 (GenBank accession no. JX258655), identified first from *Salmonella enterica* serovar Heidelberg in the United States (22), is supposed to be an ancestor of the two *mcr-1*/*mcr-2*-bearing plasmids described above (Fig. 1B).

There is awareness that the transposition of antibiotic resistance genes is a widespread genetic event catalyzed by specific mobile elements (either an insertion sequence [IS] or integron). As expected, insertion sequence IS*Apl1*, originally found in *Actinobacillus pleuropneumoniae*, is located upstream of the *mcr-1* gene in the IncI2-type *mcr-1*-harbouring plasmid pHNSHP45 (12, 13). However, the IS*Apl1* element is consistently absent in front of the *mcr-1* gene on the most IncX4 plasmids (23, 24), implying diversified conjugation-aided mechanisms for the transfer of the plasmid-borne *mcr-1* gene. In agreement with most of the *mcr-1*-harbouring IncX4 plasmids, the plasmid pESTMCR is also featured, with 2,600 bp of *mcr-1* cassette whose 3′ end consistently is a hypothetical phosphoesterase-encoding gene (*hp*) (25) (Fig. 1B). The relic of a transposition event in which a proposed inverted repeat right sequence (IRR2 [TTTTTAAGAAGGGTGAACAAGTTTAAT]) of IS*Apl1* remains at the 3′ end of the *hp* gene described above (Fig. 1C) allowed us to believe that IS*Apl1* might be involved in the transposition of *mcr-1* and then lost. In contrast, two predicted IS*1595*-like insertion sequences (Fig. 1A and B) surround the *mcr-2* gene in the IncX4 plasmid pKP37-BE, despite the lack of the insertion sequence IS*Apl1* (19). In brief, the IS*1595*-like element (714 bp) carries a transposase gene (654 bp) flanked by two inverted repeats (18 bp each) (Fig. 1B). Also, the transposase-encoding gene is similar to a fragment (bp 1531602 to 1532255) from *Moraxella bovoculi* strain 58069 (GenBank accession no. CP011374) with 75% identity and 100% query coverage, posing the possibility that it might be the origin of *mcr-2* (19). In addition to the duplicated target sites (8 bp each), 176 bp of spacer was also proposed to be present between the two probable kinds of insertion events (Fig. 1B), implying a possible hot integration site/region for the IncX4 plasmids (26). This speculation will require further experimental evidence in the near future.

**FIG 2:**
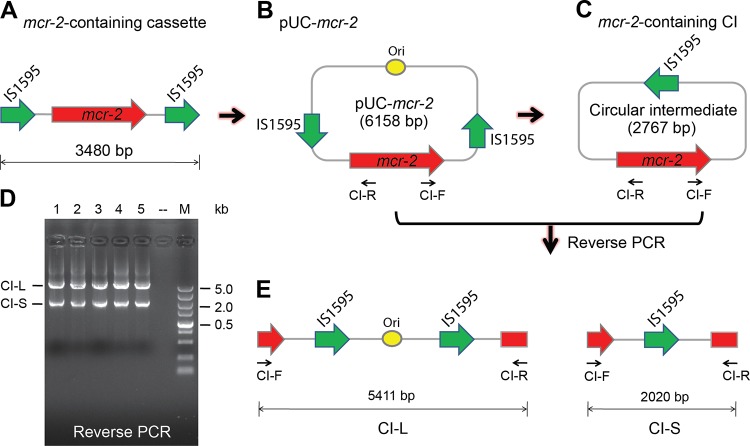
Experimental evidence for the presence of the *mcr-2*-containing circularized intermediate. (A) Scheme for the *mcr-2*-containing cassette. (B) Recombinant plasmid pUC-*mcr-2* carrying this *mcr-2*-positive cassette. The *mcr-2* gene neighbor with IS*1595*-like sequences (3,480 bp) is synthesized *in vitro* and cloned into the pUC57 vector, giving recombinant plasmid pUC-*mcr-2*. (C) Illustration of the *mcr-2*-containing circularized intermediate, which is derived from the engineered plasmid pUC-*mcr-2* in *E. coli*. (D) Reverse PCR assay for the circularized intermediates. A colony of *E. coli* carrying the pUC-*mcr-2* acts as the template for the PCR system. Two types of amplicons are acquired, namely, CI-L (the large circularized intermediate), and CI-S (the small circularized intermediate). The numbers 1, 2, 3, 4, and 5 denote five individual repeats of reverse PCR experiments. Minus indicates the blank control in which no template is added. (E) Diagram of the two types of amplicons in reverse PCR assays.

It seems likely that unlike the IS*Apl1*-mediated transposition of *mcr-1*, a unique IS*1595*-containing composite transposon enables the acquisition/transfer of the *mcr-2* gene (Fig. 1B). Moreover, the *mcr-2* colistin resistance gene with two identical IS elements might be enabled to form a circularized intermediate by homologous recombination of the IS elements (27). To test this hypothesis, we synthesized a cassette in which the *mcr-2* gene is flanked by IS*1595* on both sides (Fig. 2A) and cloned into the vector pUC57, giving the plasmid pUC57-*mcr-2* (Fig. 2B). Following introduction into the recipient *E. coli*, the *mcr-2*-containing intermediate might be generated (Fig. 2C). As anticipated, two types of specific amplicons were produced in the reverse PCR assays (Fig. 2D and E), which were verified by direct DNA sequencing. The reverse PCR results demonstrated the presence of *mcr-2*-containing circularized intermediates, implying the IS*1595*-like element was involved in the mobilization of *mcr-2*.

Therefore, we anticipated that the appearance of a circularized intermediate might accelerate the fast spread/dissemination of MCR-2 colistin resistance among diversified bacterial hosts. Together, we proposed that the transposal mechanism for the acquisition/transfer of the *mcr-2*-containing antibiotic cassette is far different from that of *mcr-1* in the IncX4 plasmid (Fig. 1B).

### Phylogeny of MCR-2.

**FIG 3:**
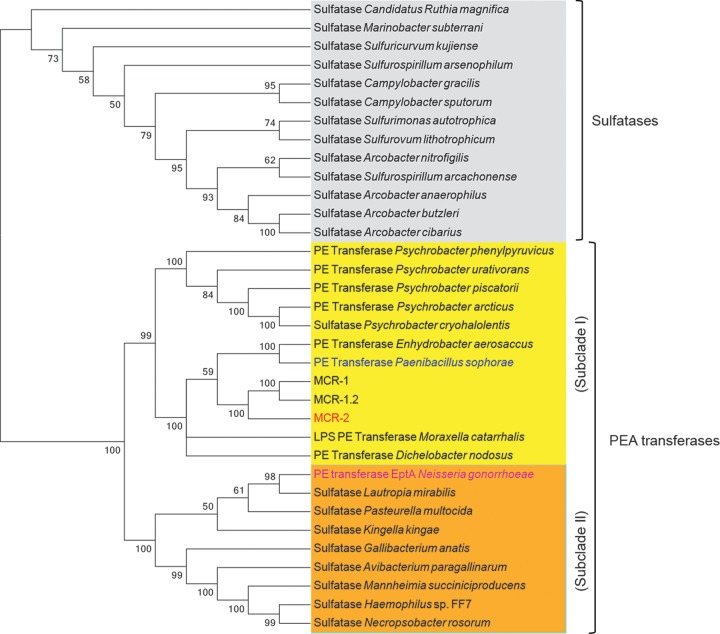
Phylogeny of the MCR-2 and its homologues. Evolutionary history was inferred by using the maximum likelihood method based on the 2008 model of Le and Gascuel (53). The bootstrap consensus tree inferred from 1,000 replicates (54) is taken to represent the evolutionary history of the taxa analyzed (54). Branches corresponding to partitions reproduced in less than 50% of bootstrap replicates are collapsed. The percentages of replicate trees in which the associated taxa are clustered in the bootstrap test (1,000 replicates) are shown next to the branches (54). Initial trees for the heuristic search were obtained automatically by applying neighbor-joining (NJ) and BioNJ algorithms to a matrix of pairwise distances estimated using a JTT model and then selecting the topology with superior log likelihood value. A discrete gamma distribution was used to model evolutionary rate differences among sites: 5 categories (+*G*), parameter = 1.5280. The rate variation model allowed for some sites to be evolutionarily invariable (+*I*; 10.3586% of sites). The analysis involved 34 amino acid sequences. All positions with less than 95% site coverage were eliminated. That is, fewer than 5% alignment gaps, missing data, and ambiguous bases were allowed at any position. There were a total of 511 positions in the final data set. Evolutionary analyses were conducted in MEGA7 (29). It seems likely that the phylogeny carries two groups: sulfatases (in gray) and phosphoethanolamine transferases (PEA transferases). The latter consisted of two subclades: subclade I (in yellow), including MCR-2 (in red) and the PEA transferase of *Paenibacillus sophorae* (in blue), and subclade II (in orange) with the *Neisseria gonorrhoeae* LptA (in purple) included.

A blastp search (with a cutoff of >30% identity) with MCR-2 and/or MCR-1 as the primer sequence returned a collection of related homologues with the annotation of either PEA transferases or sulfatases (28). As a result, we retrieved 34 protein sequence candidates. The phylogeny of the MCR-2 protein constructed by MEGA7 (29) presented clearly two distinct groups: one group denotes a family of PEA transferases containing MCR-2, MCR-1, and *Neisseria* LptA (Fig. 3), whereas the other group comprises a series of putative sulfatases (Fig. 3). Of particular note, it seems likely that two subclades (I and II) can be assigned to the family of PEA transferases. Subclade I was featured with the plasmid-borne MCR-2 (and/or MCR-1) and the chromosomally encoded *Paenibacillus* PEA transferase (30–32) (Fig. 3). In contrast, subclade II was associated with the chromosomally encoded *Neisseria* LptA (Fig. 3). Obviously, this might suggest divergence in the evolutional patterns of the LptA/MCR-2 integral membrane proteins. In particular, MCR-2 and LptA can be functionally equivalent partially (if not all), despite the fact they are grouped into two distinct subclades (Fig. 3). However, we cannot exactly access the ancestor for the MCR-2 protein right now because that experimental evidence is not sufficient.

**FIG 4:**
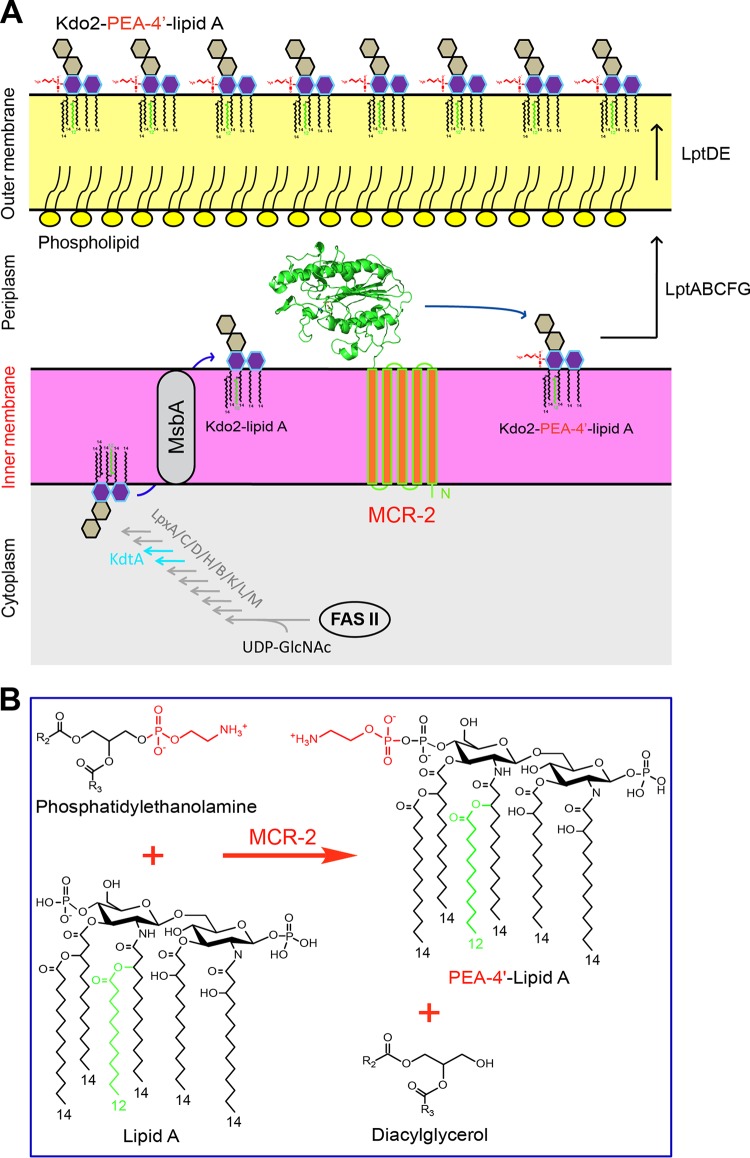
Metabolic mechanism of MCR-2-mediated colistin resistance. (A) Schematic representation for LPS-lipid A modification by MCR-2 in *E. coli*. In the cytoplasm, bacterial LPS-lipid A is synthesized using UDP-GlcNAc as the primer substrate. The fatty acid intermediates (C_12_ and C_14_) from the bacterial type II fatty acid synthesis (FAS II) pathway enter into the conservative 10-step route of lipid A synthesis involving nine enzymes (LpxA, LpxC, LpxD, LpxH, LpxB, LpxK, LpxL, LpxM, and KdtA). The nascent lipid A from the cytoplasm is translocated by the ABC transporter MsbA, a lipid flippase (35), across the inner membrane into the periplasm. The integral membrane protein MCR-2 is supposed to be localized on the periplasm side of inner membrane and catalyzes the chemical modification of the 2-keto-3-deoxyoctulosonic acid (Kdo2)-lipid A, giving Kdo2-PEA-4′-lipid A. The modified form of Kdo2-lipid A, Kdo2-PEA-4′-lipid A, then is exported by LptABCFG and LptDE into the outer leaflet of the outer membrane (36), thus reducing the negative membrane charge. That is the reason for the low/decreased affinity of bacterial surface to the cationic antibiotic polymyxin. (B) Chemical reaction in which MCR-2 catalyzes the modification of lipid A with 4′-phosphatidylethanolamine. MCR-2 catalyzes the addition of phosphatidylethanolamine to position 4′ of lipid A, giving the final products of both PEA-4′-lipid A and diacylglycerol. This was adapted from our model proposed for MCR-1 (40) with minor modification. The molecular structures were drawn using the ChemDraw software.

**FIG 5:**
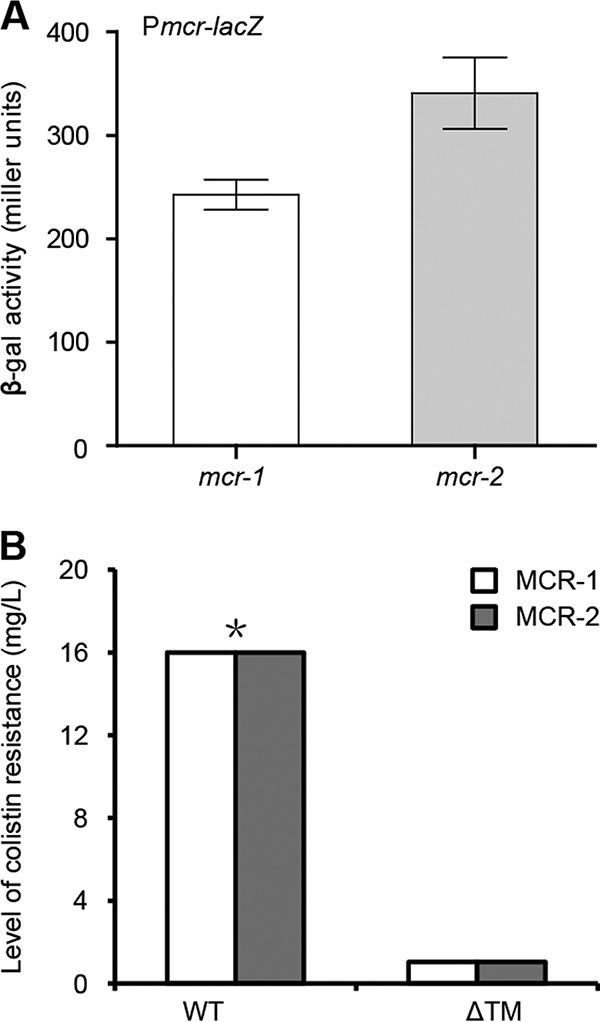
Transcriptional and functional analyses of the MCR-2 protein. (A) LacZ-based measurement of the *mcr-2* promoter activity. To measure bacterial β-galactosidase (β-Gal) activity, log-phase cultures of *E. coli* MC4100 carrying the transcriptional fusions of either P*mcr-1*-*lacZ* or P*mcr-2*-*lacZ* (Table S1) were sampled. Data are collected from three independent experiments and expressed as average ± standard deviation (SD). The data suggested that the transcriptional level of the *mcr-2* promoter is appreciably higher than that of the *mcr-1*. (B) Removal of the transmembrane regions (TM1 of MCR-1 and TM2 of MCR-2) impairs its role in colistin resistance by MCR-1 and/or MCR-2. The two wild-type (WT) strains denote the *E. coli* MG1655 strain carrying either pBAD24::*mcr-1* or pBAD24::*mcr-2*. The ΔTM mutant includes ΔTM1 (the *mcr-1* mutant with deletion of N-terminal transmembrane region from bp 1 to 540) and ΔTM2 (the *mcr-2* mutant with deletion of the N-terminal transmembrane region from bp 1 to 534), respectively. The method culture dilution on LBA plates was used, and a representative result from three independent experiments is given. An asterisk indicates the growth of the recipient *E. coli* strain that express the *mcr-2* gene is appreciably better than that of the counterpart expressing the *mcr-1* gene under the condition with 16 mg/liter of colistin.

Domain analyses of MCR-2, a 538-amino-acid (aa) polypeptide, defines it as consisting of a transmembrane region connected to a sulfatase domain (see Fig. S2A in the supplemental material). As a core domain having the ability to hydrolyze a sulfate group, sulfatase is distributed extensively in three domains of life (33, 34). Because deletion of the transmembrane domain (ΔTM2) impairs fully the phenotype in the colistin resistance conferred by MCR-2 (see Fig. 5), we thus hypothesize that the acquisition of a transmembrane domain enables the PEA transferase to localize correctly in the periplasm (Fig. 4) and then modify possible substrates like lipid A. Finally, the situation of MCR-2 in the phylogenetic tree allowed us to believe that a potentially parallel evolutionary path can be assigned to the two cousins (MCR-2 and LptA) under some environmental selection pressures (especially the extensive use of colistin as a veterinary/clinical medicine) (Fig. S2).

10.1128/mBio.00625-17.2 FIG S2  Bioinformatics analyses of the MCR-2 integral membrane protein. (A) TMHMM-aided prediction of the transmembrane helices in proteins suggests that the MCR-2 protein is a putative integral membrane protein with five transmembrane segments at the N terminus. The polypeptide sequence of MCR-2 was subjected to TMHMM server v2.0 (http://www.cbs.dtu.dk/services/TMHMM/) for prediction of the topological structure. (B) Sequence comparison of the MCR-2 protein and its homologues by multiple sequence alignment was conducted using Clustal Omega (http://www.ebi.ac.uk/Tools/msa/clustalo/), and the final output was given via the process used by the program ESPript 3.0 (http://espript.ibcp.fr/ESPript/cgi-bin/ESPript.cgi) (52). Identical residues are shown as white letters with a red background, similar residues are shown as red letters with a white background, and variant residues are shown as black letters. The six important residues critical for either Zn^2+^ binding or substrate binding are labeled with green arrows. Designations: TM, transmembrane; MCR-1.2, a variant of MCR-1 with only a point mutation of Q3L (55); MCR-2, a putative phosphoethanolamine transferase with 81% identity to that of MCR-1 (19). Download FIG S2, TIF file, 5.3 MB. Copyright © 2017 Sun et al. 2017 Sun et al. This content is distributed under the terms of the Creative Commons Attribution 4.0 International license.

### The periplasmic location of MCR-2 is a prerequisite for its activity.

The synthesis of LPS-lipid A begins with UDP-GlcNAc as the primer substrate in the cytoplasm of *E. coli* (Fig. 4A). Subsequently, type II fatty acid synthesis (FAS II) provides the intermediates of fatty acid (C_12_ to C_14_) that enter the lipid A synthesis pathway involving nine enzymes (namely, LpxA, LpxC, LpxD, LpxH, LpxB, LpxK, LpxL, LpxM, and KdtA in Fig. 4A). Once being flipped from the cytoplasm by the ABC transporter MsbA into the periplasm (35), the nascent lipid A can be modified by the membrane-anchoring enzyme MCR-2, giving the final product PEA-4′-lipid A (Fig. 4A and B). The replacement of lipid A with the PEA-4′-lipid A exported onto the outer leaflet of bacterial outer membrane significantly reduces the negative membrane charge (36), which consequently results in the decreased affinity of the surface in binding to colistin, the cationic polypeptide antibiotic (i.e., colistin resistance) (Fig. 4A). Therefore, it is reasonable that the subcellular location of the MCR-2 (and/or MCR-1) protein in bacterial periplasm is a prerequisite for its catalytic activity and colistin resistance. However, this requires further experimental evidence. Also, it is of much interest to compare the transcription profiles between *mcr-2* and *mcr-1*. Thus, we applied LacZ-based transcriptional analyses to the *mcr-2* gene. Unexpectedly, we noted that the *mcr-2* promoter is appreciably stronger than that of the *mcr-1* gene (Fig. 5A).

10.1128/mBio.00625-17.6 TABLE S1  Bacteria and plasmids used in this study. Download TABLE S1, DOCX file, 0.1 MB. Copyright © 2017 Sun et al. 2017 Sun et al. This content is distributed under the terms of the Creative Commons Attribution 4.0 International license.

**FIG 6:**
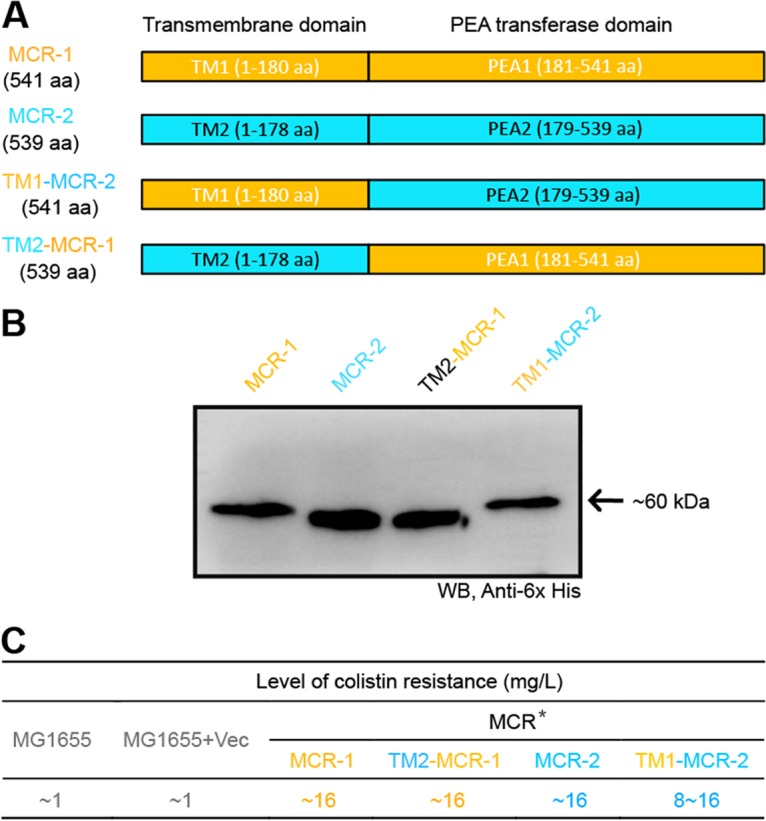
Domain-swapping-based functional dissection of the two domains (TM and PEA) of both MCR-1 and MCR-2. (A) Diagram of MCR-1 and MCR-2 and the chimeric versions TM1-MCR-2 and TM2-MCR-1. (B) Western blot analyses of expression of MCR-1 and MCR-2 and their derivatives (TM1-MCR-2 and TM2-MCR-1). The four versions of recombinant membrane protein MCR with a C-terminal 6×His tag were prepared using the *in vitro E. coli* expression system, separated by 12% SDS-PAGE, and detected by Western blotting (WB) with anti-6×His antibody as primary antibody. (C) Functional evaluation of domain swapping between the MCR-1 and MCR-2. The assays of colistin resistance were conducted using the LBA plates supplemented with colistin in a series of dilutions. The result that appears consistently in our trials is shown. An asterisk indicates that although expression of both the *mcr-1* and *mcr-2* genes can support the growth of colistin-susceptible *E. coli* strain MG1655 under the nonpermissive condition of 16 mg/liter of colistin, the latter gives appreciably better colony growth on the LBA plate.

Bioinformatic analyses indicated that MCR-2 is an integral membrane protein with N-terminal 5 transmembrane helices (Fig. S2A). Using the arabinose-inducible *E. coli* expression system pBAD24/MG1655, we probed the *in vivo* role of the transmembrane region in MCR-1 function. First, we engineered a deletion mutant of the *mcr-2* gene (ΔTM2) (Fig. 5B). Second, the method of domain swapping was applied to generate two chimeric versions of the colistin resistance gene (namely, TM1-*mcr-2* and TM2-*mcr-1*). “TM1-*mcr-2*” denotes the altered *mcr-2* gene where the transmembrane region TM2 (bp 1 to 534) is replaced with the counterpart TM1 (bp 1 to 540) of the *mcr-1* gene. In contrast, “TM2-*mcr-1*” represents the chimeric form of *mcr-1* whose extracellular transferase domain is fused to the 3′ end of the transmembrane region TM2 (bp 1 to 534) of the *mcr-2* gene (Fig. 6A).

In our assays, the negative-control strain (MG1655 with/without the pBAD24 vector) can only grow on the Luria-Bertani agar (LBA) plate with less than 1 to 2 mg/liter of colistin, whereas the positive-control strain MG1655 with the pBAD24-borne expression of the *mcr-2* in the wild type, exhibits significant growth on LBA medium with 16 mg/liter of colistin (Fig. 5B). Despite the fact that the growth of *E. coli* expressing both *mcr-1* and *mcr-2* appears on the LBA plates with colistin at 16 mg/liter (Fig. 5B), MCR-2 can confer to the recipient *E. coli* strain growth phenotype that is quite a bit better (see Fig. S3 in the supplemental material). Unlike the scenario seen with the positive control carrying the wild-type *mcr-2* gene, we noticed that the *E. coli* strain with the expression of the *mcr*-*2* mutant lacking its transmembrane region (ΔTM2) consistently fail to grow on LBA plates with over 1 to 2 mg/liter of colistin (Fig. 5B). As anticipated, the transmembrane domain-swapping experiments (TM1 and TM2) also proved the observations presented above. First, Western blotting illustrated that four *mcr* genes (*mcr-1*, *mcr-2*, and two chimeric genes [TM1-*mcr-2* and TM2-*mcr-1*]) can be expressed at an appreciable level in *E. coli* (Fig. 6B). More importantly, functional assays of domain swapping between *mcr-1* and *mcr-2* showed that the presence of the resultant two chimeric genes (TM1-*mcr-2* and TM2-*mcr-1*) can confer significant resistance of the colistin-susceptible strain MG1655 to up to 16 mg/liter of colistin (Fig. 6C).

10.1128/mBio.00625-17.3 FIG S3  Comparative analyses for the level of different *E. coli* expressing *mcr-1* (*mcr-2* and/or the chimeric forms) in the colistin resistance. To test the level of *E. coli* in colistin resistance, the log-phase cultures (OD_600_, ~1.0) in serial dilution were spotted onto LBA plates with different levels of colistin (0, 0.5, 1.0, 2.0, 4.0, 8.0, 16.0, and 32.0 mg/liter) and 0.2% arabinose. The LBA plates were maintained over 20 h at 37°C. The expression of *mcr* is triggered by the presence of arabinose. Regardless of the empty vector pBAD24, *E. coli* MG1655, a colistin-susceptible recipient strain, acts as a negative control. Designations: Vec, pBAD24; TM1-*mcr-2*, a genetically modified *mcr-2* gene whose transmembrane region, TM2, is replaced with the counterpart, TM1, of the *mcr-1* gene; TM2-*mcr-1*, a modified version of the *mcr-1* gene in which transmembrane region TM1 is replaced with TM2 of the *mcr-2* gene. Given the fact that *E. coli* MG1655 carrying the *mcr-2* gene (and/or TM2-*mcr-1*) grows better than the counterpart with *mcr-1* (and/or TM1-*mcr-2*), it seems likely that the transmembrane region TM2 might be more efficient than TM1 in facilitating the correct localization of MCR in the periplasm. Download FIG S3, TIF file, 5 MB. Copyright © 2017 Sun et al. 2017 Sun et al. This content is distributed under the terms of the Creative Commons Attribution 4.0 International license.

**FIG 7:**
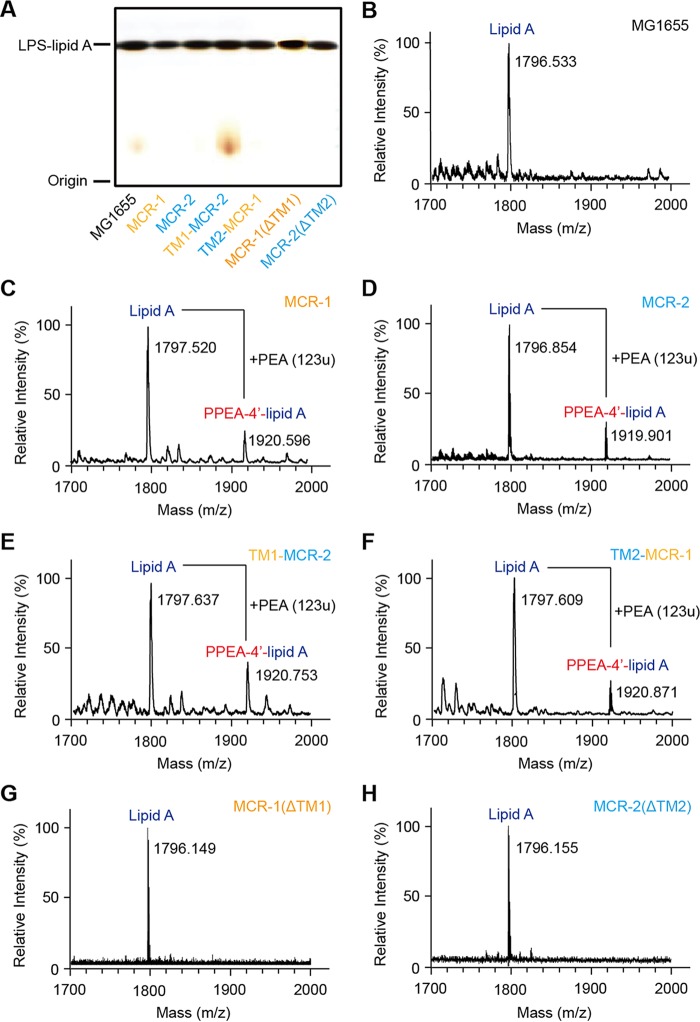
MALDI-TOF MS-based examination of the LPS-lipid A modifications by the MCR-1 and MCR-2 enzymes and their derivatives. (A) Silver-staining analyses of the isolated *E. coli* lipopolysaccharide (LPS) containing lipid A (LPS-lipid A). The bacterial LPS was isolated routinely from five *E. coli* species, including MG1655 with MCR-1 and/or MCR-2, TM2-MCR-1, TM1-MCR-2, MCR-1(ΔTM1), and MCR-2(ΔTM2) (Table S1) or MG1655 alone. The LPS was then separated by 12% PAGE and visualized by silver staining. Following further purification, the acquired lipid A with saccharide residues removed was subjected to MALDI-TOF MS-based analyses. (B) MS profile of the lipid A isolated from the negative-control strain, *E. coli* MG1655. (C) MS analyses of the lipid A for *E. coli* MG1655 expressing MCR-1. (D) MS-based assays for the lipid A for *E. coli* MG1655 expressing MCR-2. (E) MS-aided detection of the altered lipid A profile from *E. coli* MG1655 upon expression of the chimeric version TM1-MCR-2. (F) MS-based visualization for the modified version of the lipid A from *E. coli* MG1655 in the presence of TM2-MCR-1. (G) MS-based evidence for absence of the lipid A modified in *E. coli* MG1655 carrying the MCR-1(ΔTM1) deletion mutant. (H) MS-based assay for the lipid A profile from *E. coli* MG1655 with the nonfunctional version of MCR-2, MCR-2(ΔTM2). In principle, the *bis*-phosphorylated hexa-acylated lipid A (*m*/*z* ~1,797) appears in the negative-control strain *E. coli* MG1655, whereas the MG1655 strain with the expression of the *mcr-1*/*mcr-2* gene or its derivatives was consistent with one PEA added to the *bis*-phosphorylated structure (*m*/*z* ~1,920; i.e., 1,797 + 123). Similar to the scenario seen with the negative-control MG1655 strain, the peak of lipid A appears at *m*/*z* 1,796.149 [for the MCR-1(ΔTM1) mutant] and at *m*/*z* 1,796.155 [for the MCR-2(ΔTM2) mutant], respectively. As anticipated, MS detection allowed us to visualize the appearance of a unique peak at the positions of masses (1,919.901 to ~1,920.871) with various intensities upon the expression of MCR-2/MCR-1 and derivatives. The result provided metabolic evidence that MCR-2/MCR-1 catalyzes the modification of lipid A by an addition of PEA (Fig. 4). The bacterial LPSs were isolated as described above.

To gain further metabolic evidence, we isolated LPS-lipid A for extensive analyses by matrix-assisted laser desorption ionization–time of flight mass spectrometry (MALDI-TOF MS) (Fig. 7A). The MS results demonstrated that unlike the negative-control MG1655 with or without empty vector pBAD24 (Fig. 7B), the lipid A modification occurs consistently in the strains carrying any one of the following four MCR versions: MCR-1 (Fig. 7C), MCR-2 (Fig. 7D), TM1-MCR-2 (Fig. 7E), or TM2-MCR-1 (Fig. 7F). Also, the engineered strains with the transmembrane deletion mutations of either MCR-1 (ΔTM1) or MCR-2 (ΔTM2) consistently lack the unique alteration of lipid A (Fig. 7G and H). Obviously, the above data represent solid evidence that the transmembrane regions TM2 and TM1 play a critical role in MCR-2-mediated colistin resistance. Therefore, we are allowed to believe that the catalytic activity of PEA transferase depends on the correct location of MCR-2 in bacterial periplasm (Fig. 4A).

### Structure-guided functional mapping of MCR-2.

To the best of our knowledge, MCR-1 (12, 13) and its newly identified variant, MCR-2 (19), are the only two known plasmid-borne genes encoding the PEA transferases so far. However, limited information is available regarding to the genetic determinant and functional aspects of MCR-2 (and/or MCR-1). We aimed to address these unanswered questions. As expected, the two MCR homologues (MCR-1 and MCR-2) are pretty conserved in the extracellular domain of PEA transferase in comparison with its cousin, LptA (EptA) of *Neisseria*. Multiple sequence alignment suggests that a putative zinc-binding/catalytic motif comprising six conservative residues (E244, T283, H393, D463, H464, and H476) is present in MCR-2 (Fig. S2B). Of note, the locations of the critical residues in MCR-1 denote E246, T285, H395, D465, H466, and H478, respectively (Fig. S2B).

**FIG 8:**
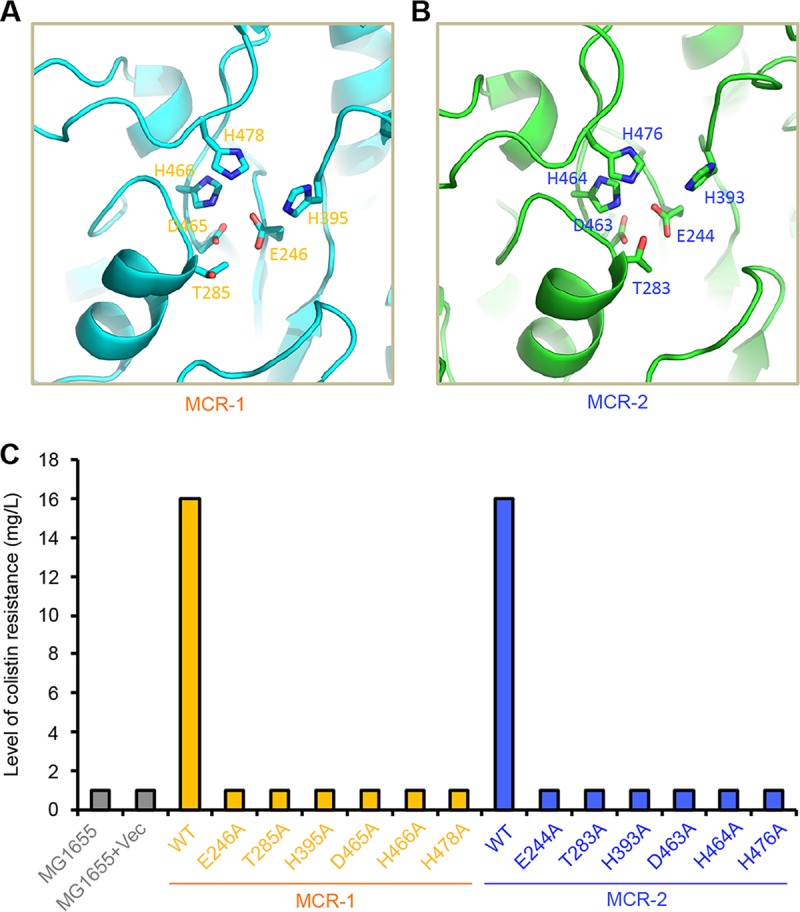
Structure-based mapping of the functional motif for MCR-2. (A) Structural visualization of the six residues critical for the activity of MCR-1 PEA-lipid A transferase. (B) Structural insights into the catalytic motif comprising six residues in the MCR-2 PEA-lipid A transferase. The ribbon structures of both MCR-1 and MCR-2 were modeled by the Swiss-Model program with *Neisseria* LptA (PDB no. 4KAV) (18) as a structural template and were generated via the PyMol software. In addition to the five essential residues (E246, T285, H395, D465, and H466) we recently determined, one more key residue, H478, has been assigned to the MCR-1 function. In the MCR-2 protein, the critical motif included the following six residues: E244, T283, H393, D463, H464, and H476. (C) Site-directed mutagenesis-based assay for the functional motif of MCR-2. The *mcr-2* point mutants were assayed using LBA plates with colistin in a series of dilutions. A representative result from our trials is shown. The negative control denotes MG1655 alone (or with empty vector). Vec, vector pBAD24.

Using membrane protein expression technology, we attempted to prepare the four MCR proteins (MCR-1, MCR-2, TM1-MCR-2, and TM2-MCR-1 in Fig. S4A to D in the supplemental material) and purified them to homogeneity (Fig. S4A to D). Size exclusion analyses suggested that they both can form in a monomer *in vitro* (Fig. S4A to D). Besides the confirmation by Western blotting using an anti-6×His primary antibody (Fig. 6B), MS-based determination validated the molecular mass and its identity for the recombinant MCR-2 transmembrane protein (see Fig. S5A and B in the supplemental material). To gain further structural insight into the catalytic mechanism of MCR-2 (i.e., the addition of PEA to 4′-phosphate of lipid A from phosphatidylethanolamine), structural modeling by the Swiss-Model program was performed using *Neisseria* LptA (PDB accession no. 4KAV) (18) as a structural template (Fig. 8). The ribbon structures of MCR-2 (Fig. 8B) together with MCR-1 (Fig. 8A) were generated with the PyMol software. Intriguingly, structural comparison of the two MCR proteins illustrated that the putative 6 conserved residues (Fig. S2B) do constitute almost identical motifs (Fig. 8A and B). Thus, we are extremely interested in examining their physiological role in the MCR-2 catalytic activity-dependent colistin resistance phenotype.

10.1128/mBio.00625-17.4 FIG S4  Biochemical characterization of four versions of the integral membrane protein MCR. (A) Fast protein liquid chromatography (FPLC) profile of the MCR-1 membrane protein. (B) Size exclusion analyses of the MCR-2 protein. (C) FPLC assay for the chimeric TM1-MCR-2 protein. (D) Size exclusion analyses of the chimeric TM2-MCR-1 protein. FPLC assays were conducted with a Superdex 75 column. Abbreviations: mAU, milliabsorbance units; M, protein molecular mass marker (in kilodaltons). The elution volume of MCR-1, MCR-2, TM1-MCR-2, and TM2-MCR-1 is at the position of ~9.5 ml and gives an estimated mass of 60 to ~70 kDa, which matches well its ideal mass of ~60 kDa. The inset gels are 12% SDS-PAGE profiles of MCR-1, MCR-2, TM1-MCR-2, and TM2-MCR-1. The results suggest that MCR-1 and MCR-2 exhibit the solution structure of a monomer *in vitro*. Download FIG S4, TIF file, 1.4 MB. Copyright © 2017 Sun et al. 2017 Sun et al. This content is distributed under the terms of the Creative Commons Attribution 4.0 International license.

10.1128/mBio.00625-17.5 FIG S5  MS verification of the MCR-2 protein. (A) MALDI-TOF MS determination of the mass of the MCR-2 protein. (B) MS validation of the MCR-2 polypeptide. The identified polypeptide is highlighted by boldface and underlined letters. Download FIG S5, TIF file, 0.9 MB. Copyright © 2017 Sun et al. 2017 Sun et al. This content is distributed under the terms of the Creative Commons Attribution 4.0 International license.

Driven by the above structural and bioinformatics speculation, we applied site-directed PCR mutagenesis to generate the following 6 point mutations of *mcr-2* (E244A, T283A, H393A, D463A, H464A, and H476A). Similarly, six point mutations of *mcr-1* were also used here. In the functional assays, the expression of all the versions of MCR-2 (and/or MCR-1) was triggered by the arabinose-inducible promoter of the pBAD24 expression vector in *E. coli* MG1655. As expected, strain MG1655 with or without vector alone (negative control) cannot show any growth when colistin is supplemented at over 2 mg/liter, whereas the positive-control strain MG1655 with expression of wild-type MCR-2 (or MCR-1) can grow well under conditions with up to 16 mg/liter colistin (Fig. 8C). In contrast to the positive control, none of the six *mcr*-*2* (or *mcr-1*) point mutations can confer robust growth to the recipient strain MG1655 under conditions with over 2 mg/liter of colistin, which is similar to the finding with the negative control (Fig. 8C). The data constitute *in vivo* evidence that the six-residue-containing motif plays critical roles in the maintenance of the biochemical mechanism of MCR-2 and its phenotype of colistin resistance.

## DISCUSSION

In the Gram-negative bacteria, modifications of the LPS-lipid A moiety might reduce the net negative charge of the bacterial outer membrane, which consequently impairs the attachment of cationic antimicrobial peptide (CAMP), like polymyxins (Fig. 1B; Fig. S1A), to the bacterial surface (37, 38). A total of three types of chemical modifications of the lipid A have been identified to account for bacterial resistance to colistin (37, 38). They included (i) addition of phosphoethanolamine to 4′-phosphate position of sugar originally found in *K. pneumoniae* (11), (ii) modification of sugar with aminoarabinose at the 4′-phosphate position in *Cupriavidus metallidurans* (38), and (iii) glycine (and/or diglycine) modification at 3′-linked secondary acyl chain of lipid A in *Vibrio cholerae* El Tor (37). A single enzyme, ArnT, modifies the lipid A with aminoarabinose in the periplasm of *C. metallidurans* (38), whereas, a three-protein system is required for the modification of the lipid A with glycine (and/or diglycine) in the cytoplasm of *V. cholerae* El Tor, comprising (i) glycine carrier protein AlmF (Vc1578), (ii) amino acid ligase AlmE (Vc1579), and (iii) glycine transferase AlmG (Vc1577) (37). Given the fact that the three genes (*almG*-*amlF-almE*) are acquired by gene horizontal transfer in *V. cholerae* El Tor, comprising the operon Vc1577-Vc1578-Vc1579 (37), it is reasonable to ask whether it can be transferred into closely related enterobacteria like *E. coli* and *Salmonella* and in turn promote extensive resistance to polymyxin.

MCR-1 represents the unique plasmid-borne machinery that also allows the modification of the lipid A with phosphoethanolamine at the 4′-phosphate position, thereafter conferring efficient resistance to colistin (12, 13). Unlike the chromosome-encoded enzyme ArnT which features 11 N-terminal transmembrane regions (38), the MCR-1 protein is an integral membrane protein with five periplasmic transmembrane helices (12, 13). In light of the lack of significant homology (27.1% similarity and 12.7% identity) between the two enzymes (ArnT and MCR-1), we are very interested in examining in the near future whether or not they are functionally replaced in *E. coli*. In light of the transferability of colistin resistance by the MCR-1, we are not surprised by its fast sweeping across nearly the entire world in less than 1 year since its first discovery late in 2015 (17). The results from our group together with those from other groups worldwide suggest that MCR-1 is a very conservative protein that has undergone little selective pressure.

Very recently, Xavier and colleagues reported a novel plasmid-borne gene, *mcr-2*, that also confers resistance to colistin (19), although it seems unusual that the *mcr-2* gene is detected only in Belgium and nowhere else worldwide. In fact, we and our collaborators in China attempted to perform a PCR screen for *mcr-2* using almost 10,000 animal/clinical samples. It seems likely that the absence of *mcr-2* in China might verify its low prevalence (39). This posed a hypothesis that might be due to a mechanism for *mcr-2* dissemination different from that of the paradigm *mcr-1* gene. In this report, we illustrated a working model for the transfer of the *mcr-2*-containing cassette by a unique transposon-like event (Fig. 1). The phylogeny suggested that MCR-2 and MCR-1 might share similar evolutionary history for functional acquisition of colistin resistance (Fig. 3). Consistent with that of MCR-1, the correct location of MCR-2 in periplasm ensures its enzymatic activity (Fig. 4). In addition to the five essential residues (E246, T285, H395, D465, and H466) for MCR-1 function we determined very recently (40), we demonstrated the requirement for one more residue, H478. Equivalently, we here have defined the genetic requirement of a substrate-binding/catalytic domain containing six residues (E244, T283, H393, D463, H464, and H476) for MCR-2 colistin resistance function (Fig. 6). In particular, during the review of our submitted manuscript, three reports added the X-ray structure of the catalytic domain of the MCR-1 and also added a similar structural clue to a full set of functional motifs, but it lacked full experimental verification for either MCR-1 or MCR-2 (41–43).

In summary, our findings establish mechanisms for the transfer, origin, and function of MCR-2 in transferable colistin resistance with relevance to lipid A modification and extend our understanding of a membrane charge-based remodeling strategy used for acquisition of bacterial antibiotic resistance, which might provide clues to the design of compounds targeted at reversing MCR-2/MCR-1 colistin resistance.

## MATERIALS AND METHODS

### Strains and growth conditions.

All of the bacterial strains used here are *E. coli* K-12 derivatives (see Table S1 in the supplemental material). Luria-Bertani (LB) liquid medium and LB agar (LBA) plates were used for the maintenance of *E. coli* strains. DH5α was a recipient strain for cloning of the *mcr-2* gene and its promoter, whereas strain BL21(DE3) was a protein expression host (13). DH5α(λ*-pir*) was used to propagate pAH125 and its derivatives, and MC4100 (and/or MC1061), a *lacZ* mutant strain, was subjected to producing the *mcr-2* promoter-driven *lacZ* transcriptional fusion (pAH125-P*mcr-2*) integrated on the chromosome. The colistin-susceptible strain MG1655 was utilized to assay the phenotype of MCR-2 (and/or its mutants) in resistance to colistin. All of the strains were maintained at either 37 or 30°C.

### *De novo* synthesis of the *mcr-2* gene and its promoter.

As we described earlier (44), with little change, overlapping PCR was adopted to synthesize the *mcr-2* gene at full length. Briefly, 35 pieces of DNA primers (see Table S2 in the supplemental material) were designed to amplify five overlapped DNA fragments that fully cover the *mcr-2* gene. Subsequently, the mixture of the above five PCR products diluted appropriately was subjected as the templates to the second round of overlapping PCR with a pair of specific primers (MCR-2-F and MCR-2-R [Table S2]). As a result, the *mcr-2* PCR product of the expected size was directionally cloned into the arabinose-inducible pBAD24 via the two restriction enzymes EcoRI and SalI, giving the recombinant plasmid pBAD24::*mcr-2* (Table S1). Similarly, the *mcr-2* promoter region (~300 bp) was also synthesized *in vitro* by overlapping PCR (see Tables S3 and S4 in the supplemental material) and inserted directionally into the promoter-less pAH125 with SalI and EcoRI, giving the plasmid pAH125-P*mcr-2*. All of the acquired plasmids were verified by PCR detection (Table S3) and Sanger sequencing, and the point mutations detected in the *mcr-2* gene were recovered through site-directed mutagenesis.

10.1128/mBio.00625-17.7 TABLE S2  Primers used for detection, transcription, and expression in this study. Download TABLE S2, DOCX file, 0.1 MB. Copyright © 2017 Sun et al. 2017 Sun et al. This content is distributed under the terms of the Creative Commons Attribution 4.0 International license.

10.1128/mBio.00625-17.8 TABLE S3  Primers designed for *de novo* synthesis of the *mcr-2* gene. Download TABLE S3, DOCX file, 0.1 MB. Copyright © 2017 Sun et al. 2017 Sun et al. This content is distributed under the terms of the Creative Commons Attribution 4.0 International license.

10.1128/mBio.00625-17.9 TABLE S4  Primers used for *mcr-2* promoter synthesis. Download TABLE S4, DOCX file, 0.1 MB. Copyright © 2017 Sun et al. 2017 Sun et al. This content is distributed under the terms of the Creative Commons Attribution 4.0 International license.

### Genetic manipulations.

To address the role of the transmembrane region of the *mcr-2*, a TM deletion mutant (ΔTM2) was PCR amplified and cloned into pBAD24, generating pBAD24::*mcr-2*(ΔTM2). To probe whether or not the transmembrane region (and/or extracellular PEA transferase domain) is functionally exchangeable between MCR-2 and MCR-1, the domain-swapping experiments were conducted using overlapping PCR (Table S3). Consequently, the two chimeric MCR genes TM2-*mcr-1* (in which the transmembrane region of the *mcr-2* gene is fused with the PEA transferase domain of the *mcr-1* gene) and TM1-*mcr-2* (in which the transmembrane region of the *mcr-1* gene is fused with the PEA transferase domain of the *mcr-2* gene) were generated. To finely map the catalytic domain of the MCR-2 protein, the experiments with site-directed mutagenesis were carried out as described previously (45). Besides residue H478 of MCR-1, six putative important amino acids were tested in our trials. Following confirmation of these mutant versions of MCR-2 as well as MCR-1, they were separately introduced into the recipient strain MG1655 to define the critical motifs for MCR-2 colistin resistance. In total, pBAD24 and 11 derivatives were transformed, which corresponded to pBAD24::*mcr-2*(ΔTM2), pBAD24::tm2-*mcr-1*, pBAD24::tm1-*mcr-2*, pBAD24::*mcr-1*(H478A), pBAD24::*mcr-2*, pBAD24::*mcr-2*(E244A), pBAD24::*mcr-2*(T283A), pBAD24::*mcr-2*(H393A), pBAD24::*mcr-2*(D463A), pBAD24::*mcr-2*(H464A), and pBAD24::*mcr-2*(H476A), respectively (Table S1). In addition, the MC4100 strain carrying the temperature-sensitive plasmid pINT-ts was transformed with the plasmid pAH125-P*mcr-2* to give the LacZ transcriptional fusion driven by the *mcr-2* promoter on chromosome (46).

### Expression and purification of the MCR membrane proteins.

Four versions of *mcr* genes (*mcr-1*, *mcr-2*, and two derivatives of transmembrane domain-swapping [TM1-*mcr-2* and TM2-*mcr-1*]) were separately cloned in the pET21a expression vector and transformed into *E. coli* BL21(DE3) competent cells, giving the strains FYJ915, FYJ916, FYJ917, and FYJ918, respectively (Table S1). The overnight cultures were inoculated into liquid Luria-Bertani (LB) media at the ratio of 1:100 and induced at 16°C with 0.5 mM isopropyl β-d-1-thiogalactopyranoside (IPTG) upon the optical density at 600 nm (OD_600_) reaching ~1.0 for production of the MCR proteins. Bacterial cells were harvested by centrifugation (5,000 rpm for 20 min) at 4°C and washed with 1× phosphate-buffered saline (PBS). The acquired cell pellets were resuspended in buffer A (20 mM Tris-HCl [pH 8.0], 100 mM NaCl, 5 mM DNase I, 1 mM phenylmethylsulfonyl fluoride [PMSF], 2 mM MgCl_2_) to 20% (wt/vol), lysed by a single passage through a French press (JN-Mini, China) (at 500 lb/in^2^ once and 1,300 lb/in^2^ twice), and centrifuged at 16,800 rpm for 1 h at 4°C for collection of the supernatant. Subsequently, the supernatant was subjected to spinning at 38,000 rpm for 1 h at 4°C, and the precipitant obtained was dissolved in buffer B (20 mM Tris-HCl [pH 8.0], 100 mM NaCl, 5% glycerol, 1% *n*-dodecyl-d-maltoside [DDM] [mass/vol]). Finally, the supernatant was collected after centrifugation at 38,000 rpm for 1.5 h at 4°C and incubated with preequilibrated Ni-nitrilotriacetic acid (NTA) agarose beads overnight at 4°C.

Ni-NTA agarose beads were then loaded on a column and rinsed with wash buffer (20 mM Tris-HCl [pH 8.0], 100 mM NaCl, 30 mM imidazole, 5% glycerol, 0.03% DDM [mass/vol]). The protein was eluted from the Ni-NTA agarose beads using elution buffer (20 mM Tris-HCl [pH 8.0], 100 mM NaCl, 100 mM imidazole, 5% glycerol, 0.03% DDM [mass/vol]). The eluted protein was concentrated and subsequently applied to a Resource-Q column (GE Healthcare), followed by a Superdex 75 10/300 GL size exclusion column (GE Healthcare) that was preequilibrated with 20 mM Tris-HCl (pH 8.0), 150 mM NaCl, and 0.03% DDM. The peak fractions were pooled and concentrated to approximately 10 mg/ml. The identity was judged by separation by 12% SDS-PAGE as well as Western blotting with anti-6×His primary antibody.

### Assay for colistin resistance.

As recommended by the Clinical and Laboratory Standards Institute guidelines (CLSI M100-S25), the agar dilution method was applied in colistin susceptibility experiments (47), which involves the breakpoint of EUCAST (European Committee on Antimicrobial Susceptibility Testing, 2015) for colistin resistance. The strain expressing MCR-1 refers to the positive control, whereas the MG1655 strain with/without plasmid pBAD24 alone denotes the negative control. The log-phase cultures of all the other strains carrying MCR-2 or its derivatives (Table S1) were diluted appropriately and spotted on LBA plates with colistin at various levels (0, 0.5, 1.0, 2.0, 4.0, 8.0, 16.0, and 32.0 mg/liter) (46). Of note, 0.2% arabinose was supplemented to trigger expression of MCR-2 (and/or MCR-1) (13).

### Lipid A isolation and MALDI-TOF MS.

The LPS-lipid A was routinely isolated by the method of hot phenol-water with minor change (18, 48). The *E. coli* samples were collected through stripping of colonies grown on the LBA plates with/without the addition of colistin (8 mg/liter) overnight. The resultant crude LPS was resuspended in a solution of 30 mM Tris-HCl (pH 8.0) and 0.2% SDS and subjected to (i) digestion by DNase I (25 µg/ml) and RNase A (100 µg/ml) at 37°C for 2 h followed by (ii) 1 h of incubation with proteinase K at 56°C for 1 h. After other routine purification process, the acquired LPS was loaded for 12% PAGE coupled with silver staining to judge its purity and sent out for MALDI-TOF MS-based identification as described by Hankins et al. (49).

### β-Galactosidase assays.

The two *E. coli* strains that carry the transcriptional fusion of either P*mcr-2-lacZ* or P*mcr-1-lacZ* (Table S1) were prepared for the measurement of LacZ activity. Mid-log-phase cultures grown in LB medium were directly tested after bacterial lysis with SDS-chloroform (50, 51). The data were recorded in triplicate from three independent experiments.

### Bioinformatics.

To probe its topological structure, the MCR-2 protein sequence was subjected to the TMHMM server v2.0 (http://www.cbs.dtu.dk/services/TMHMM/). The protein sequences of MCR-2 and MCR-1 were aligned using the program Clustal Omega (http://www.ebi.ac.uk/Tools/msa/clustalo/), and the final output was given after proceeding with the program ESPript 2.2 (http://espript.ibcp.fr/ESPript/cgi-bin/ESPript.cgi) (52). A blastp search with MCR-2 (and/or MCR-1) as the primer sequences was carried out to collect the related homologues with the cutoff of above 30% identity. All returned hits were annotated with either PEA transferases or sulfatases. The phylogeny of the MCR-2 protein was constructed by MEGA7 (29). Structural modeling of MCR-2 (and MCR-1) was conducted with the Swiss-Model program, in which the *Neisseria* lipooligosaccharide phosphoethanolamine transferase A (LptA) acted as a structural template (PDB accession no. 4KAV) (18), and the ribbon structure was presented with the PyMol software.
