# Supplementary figures and images for: Deciphering MCR-2 Colistin Resistance
Source: mBio. 2017 May 9;8(3):e00625-17. doi: 10.1128/mBio.00625-17 (PMC5424208; doi:10.1128/mBio.00625-17)

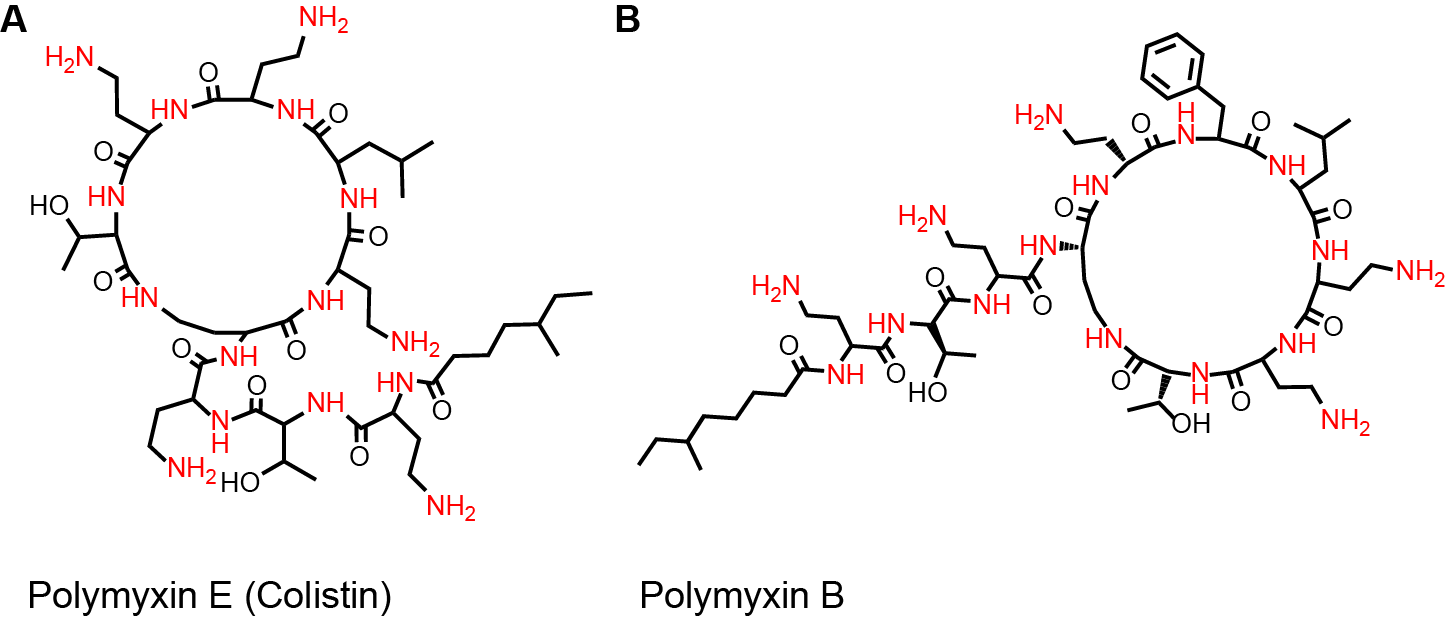

Supplement: FIG S1 [file mbo003173304sf1.tif]

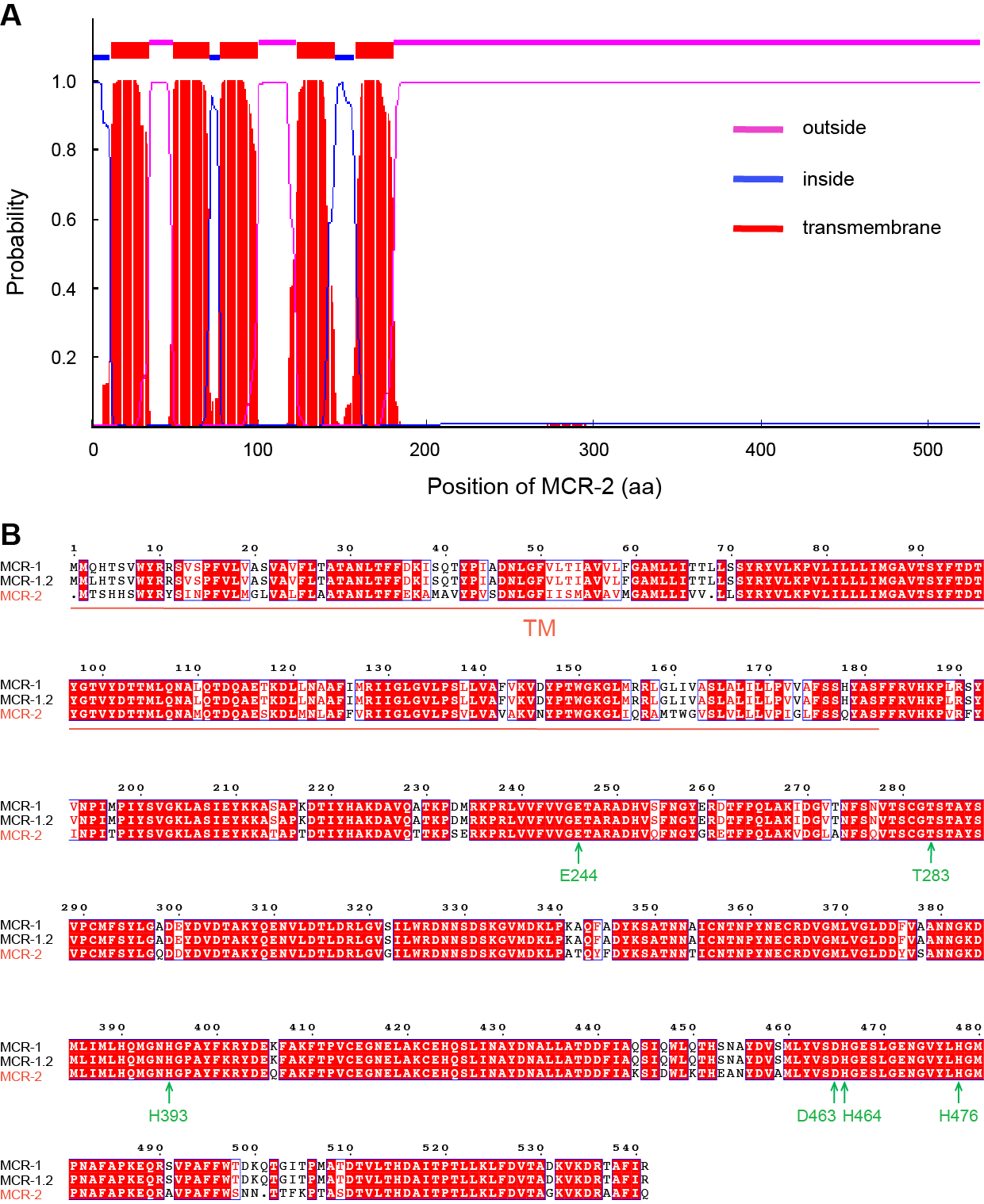

Supplement: FIG S2 [file mbo003173304sf2.tif]

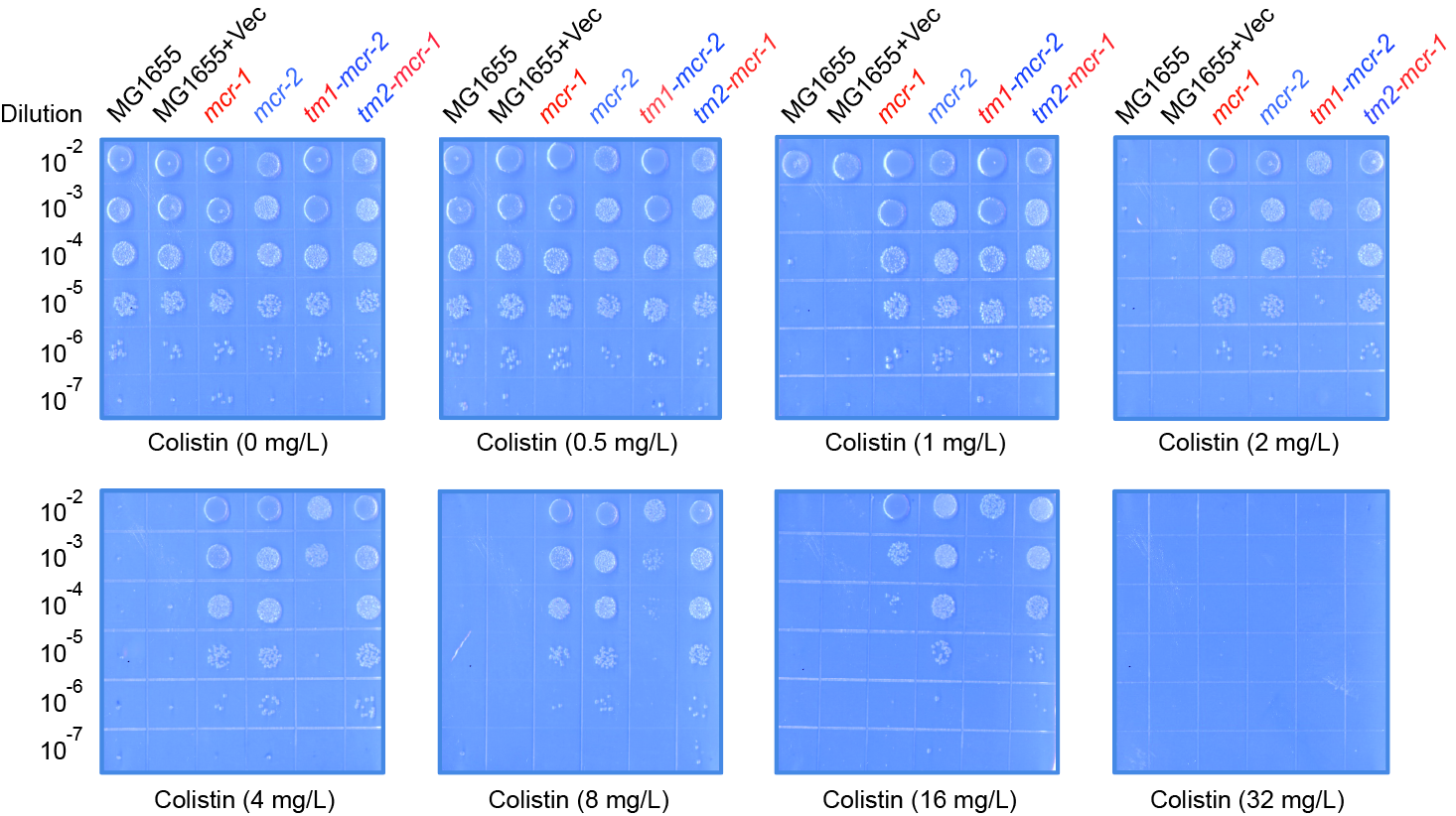

Supplement: FIG S3 [file mbo003173304sf3.tif]

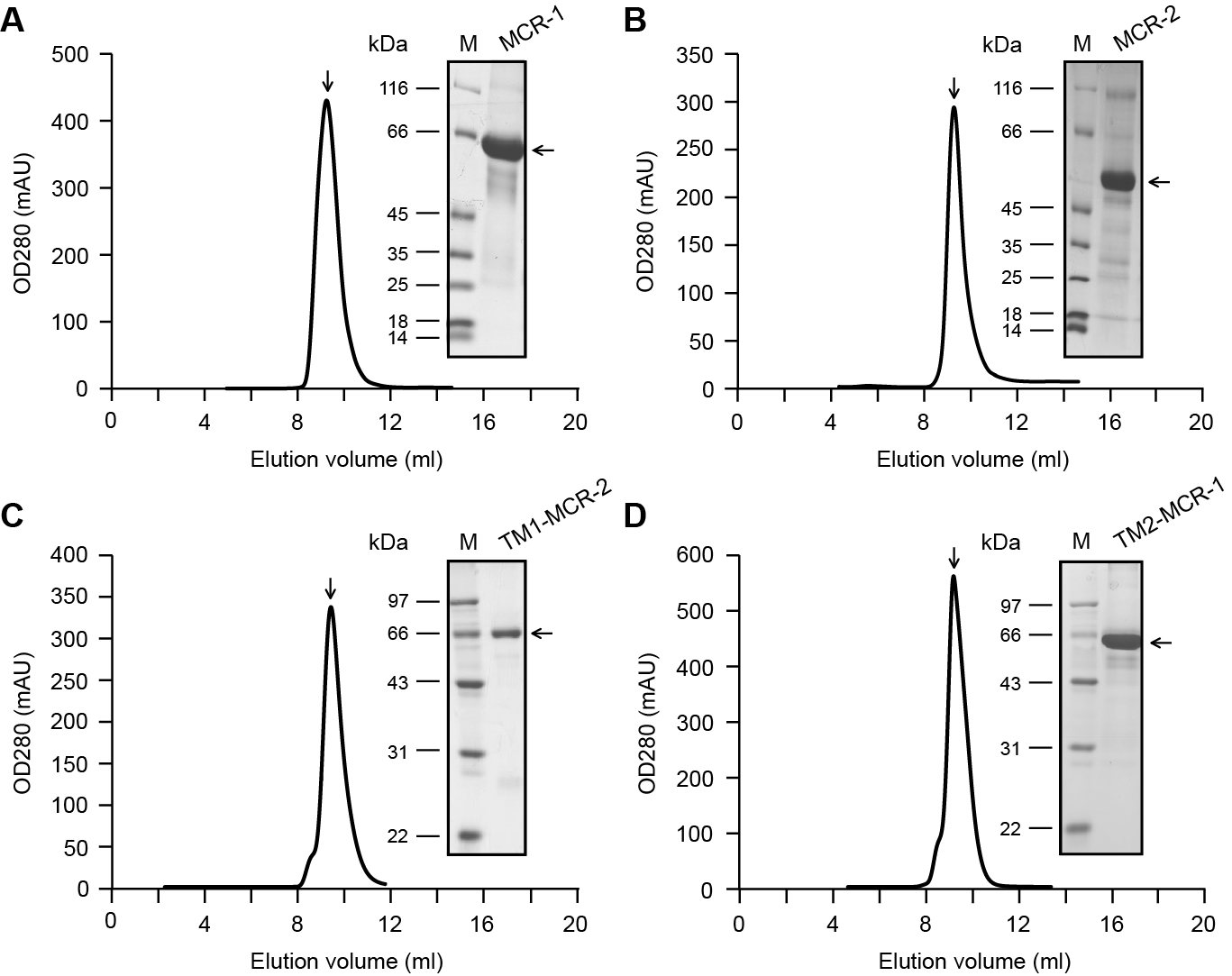

Supplement: FIG S4 [file mbo003173304sf4.tif]

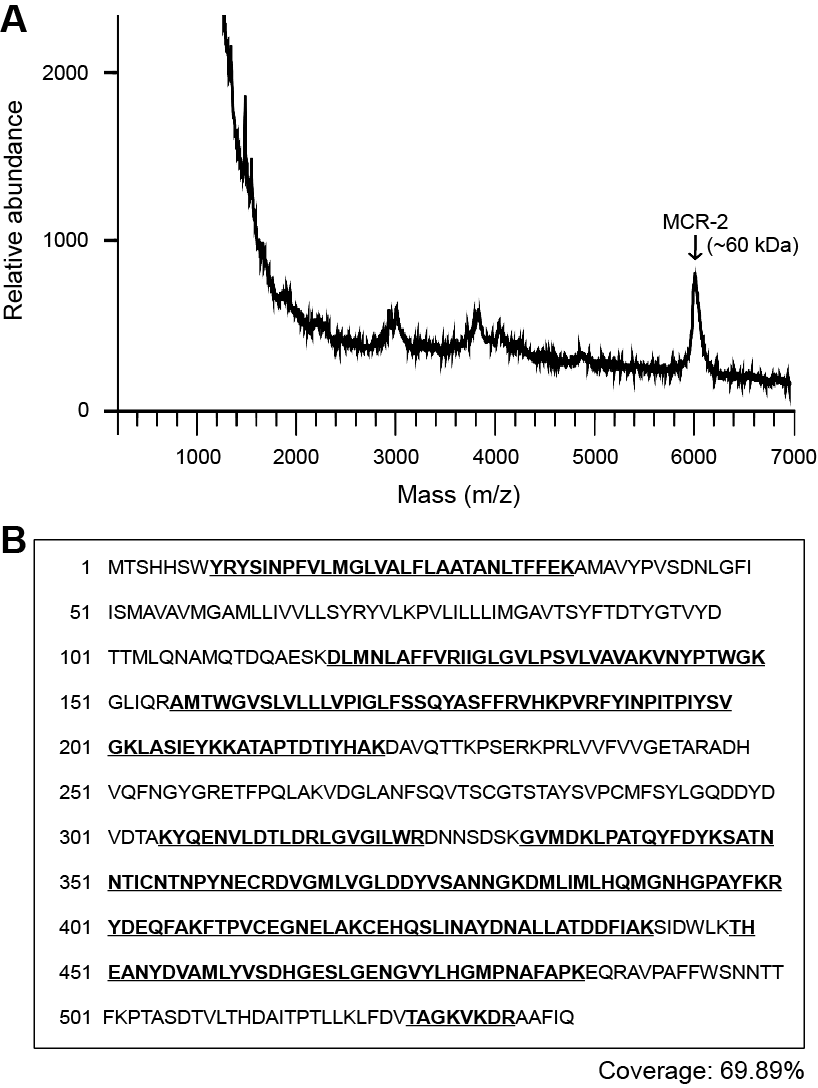

Supplement: FIG S5 [file mbo003173304sf5.tif]
