# Supplementary material for: Deciphering MCR-2 Colistin Resistance
Source: mBio. 2017 May 9;8(3):e00625-17. doi: 10.1128/mBio.00625-17 (PMC5424208; doi:10.1128/mBio.00625-17)
Supplement: TABLE S1 [file mbo003173304st1.docx]

**Table S1** Bacteria and plasmids used in this study

| Strain or plasmids | Relevant characteristics | Origins |
| --- | --- | --- |
| Strains | | |
| DH5α | Acloning host of *E.coli* | Lab stock |
| MG1655 | A wild type strain of *E.coli* | Lab stock |
| FYJ795 | MG1655 carrying pBAD24*::mcr-1* | Lab stock |
| FYJ796 | MG1655 carrying pBAD24 | Lab stock |
| FYJ833 | MG1655 carrying pBAD24*::mcr-1*(E246A) | Lab stock |
| FYJ834 | MG1655 carrying pBAD24*::mcr-1*(T285A) | Lab stock |
| FYJ835 | MG1655 carrying pBAD24*::mcr-1*(D465A) | Lab stock |
| FYJ836 | MG1655 carrying pBAD24*::mcr-1*(H466A) | Lab stock |
| FYJ837 | MG1655 carrying pBAD24*::mcr-1*(H395A) | Lab stock |
| FYJ864 | MG1655 carrying pBAD24*::mcr-1*(H478A) | Lab stock |
| FYJ850 | DH5α (λ-pir) carrying pAH125-P*mcr-1* (pE15017) | Lab stock |
| FYJ854 | MG1655 carrying PBAD24::*mcr-1*(∆TM) | Lab stock |
| FYJ855 | MG1655 carrying pBAD24::*mcr-2* | This work |
| FYJ856 | MG1655 carrying pBAD24::tm1-*mcr-2* | This work |
| FYJ857 | MG1655 carrying pBAD24::tm2-*mcr-1* | This work |
| FYJ905 | MG1655 carrying pBAD24::*mcr-2*(∆TM) | This work |
| FYJ906 | MG1655 carrying pBAD24*::mcr-2*(E244A) | This work |
| FYJ907 | MG1655 carrying pBAD24*::mcr-2*(T283A) | This work |
| FYJ908 | MG1655 carrying pBAD24*::mcr-2*(H393A) | This work |
| FYJ909 | MG1655 carrying pBAD24*::mcr-2*(D463A) | This work |
| FYJ910 | MG1655 carrying pBAD24*::mcr-2*(H464A) | This work |
| FYJ911 | MG1655 carrying pBAD24*::mcr-2*(H476A) | This work |
| FYJ912 | DH5α (λ-pir) carrying pAH125-P*mcr-2* | This work |
| FYJ913 | MC4100 with P*mcr-1*_pE15017-*lacZ* transcriptional fusion at the chromosomal attB λ site | This work |
| FYJ914 | MC4100 with P*mcr-2*-*lacZ* transcriptional fusion at the chromosomal attB λ site | This work |
| FYJ915 | BL21carrying pET21a::*mcr-1* | This work |
| FYJ916 | BL21carrying pET21a::*mcr-2* | This work |
| FYJ917 | BL21carrying pET21a::tm1*-mcr-2* | This work |
| FYJ918 | BL21carrying pET21a::tm2*-mcr-1* | This work |
| Plasmids |  |  |
| pBAD24 | Arabinose inducible promoter-driven expression vector; Amp^R^ | Lab stock |
| pBAD24::*mcr-1* | A pBAD24 carrying the wild type version of *mcr-1* at the two cuts of EcoRI and SalI; Amp^R^ | Lab stock |
| pBAD24::*mcr-1*(E246A) | pBAD24 encoding the mutant version of  *mcr-1*(E246A); AmpR | Lab stock |
| pBAD24::*mcr-1*(T285A) | pBAD24 encoding the mutant version of  *mcr-1*(T285A); AmpR | Lab stock |
| pBAD24::*mcr-1*(D465A) | pBAD24 encoding the mutant version of  *mcr-1*(D465A); AmpR | Lab stock |
| pBAD24::*mcr-1*(H466A) | pBAD24 encoding the mutant version of  *mcr-1*(H466A); AmpR | Lab stock |
| pBAD24::*mcr-1*(H395A) | pBAD24 encoding the mutant version of  *mcr-1*(H395A); AmpR | Lab stock |
| pBAD24::*mcr-1*(H478A) | pBAD24 encoding the mutant version of  *mcr-1*(H478A); AmpR | Lab stock |
| pBAD24::*mcr-2* | A pBAD24 carrying the wild type version of *mcr-2* at the two cuts of EcoRI and SalI; Amp^R^ | This work |
| pBAD24::tm1*-mcr-2* | pBAD24 encoding the transmembrane region of *mcr-1* and the extracellular of *mcr-2*; Amp^R^ | This work |
| pBAD24::tm2*-mcr-1* | pBAD24 encoding the transmembrane region of *mcr-2* and the extracellular of *mcr-1*; Amp^R^ | This work |
| pBAD24::*mcr-2*(∆TM) | pBAD24 encoding the transmembrane region deletion version of *mcr-2*; Amp^R^ | This work |
| pBAD24::*mcr-2*(E246A) | pBAD24 encoding the mutant version of  *mcr-2*(E246A); Amp^R^ | This work |
| pBAD24::*mcr-2*(T285A) | pBAD24 encoding the mutant version of  *mcr-2*(T285A) ; Amp^R^ | This work |
| pBAD24::*mcr-2*(H395A) | pBAD24 encoding the mutant version of  *mcr-2*(H395A); Amp^R^ | This work |
| pBAD24::*mcr-2*(D463A) | pBAD24 encoding the mutant version of  *mcr-2*(H463A); Amp^R^ | This work |
| pBAD24::*mcr-2*(H464A) | pBAD24 encoding the mutant version of  *mcr-2*(H464A) ; Amp^R^ | This work |
| pBAD24::*mcr-2*(H476A) | pBAD24 encoding the mutant version of  *mcr-2*(H476A); Amp^R^ | This work |
| pAH-P*mcr-1*(pE15017) | pAH125 carrying the *mcr-1* promoter region from  pE15017,Kan^R^ | This work |
| pAH-P*mcr-2* | pAH125 carrying the *mcr-2* promoter region from pKP37-BE,Kan^R^ | This work |
| pET21a::*mcr-1* | A pET21acarrying the wild type version of *mcr-1* at the two cuts of NdeI and XhoI; Amp^R^ | This work |
| pET21a::*mcr-2* | A pET21acarrying the wild type version of *mcr-2*at the two cuts of NdeI and XhoI; Amp^R^ | This work |
| pET21a::tm1*-mcr-2* | A pET21a carrying the transmembrane region of *mcr-1* and the extracellular of *mcr-2*at the two cuts of NdeI and XhoI; Amp^R^ | This work |
|  |  |  |
| pET21a::tm2*-mcr-1* | A pET21a carrying the transmembrane region of *mcr-2* and the extracellular of *mcr-1*at the two cuts of NdeI and XhoI; Amp^R^ | This work |
