# Supplementary material for: Deciphering MCR-2 Colistin Resistance
Source: mBio. 2017 May 9;8(3):e00625-17. doi: 10.1128/mBio.00625-17 (PMC5424208; doi:10.1128/mBio.00625-17)
Supplement: TABLE S2 [file mbo003173304st2.docx]

**Table S2** Primers used for detection, transcription and expression in this study

| Primers | Sequences |
| --- | --- |
| MCR-1-F (EcoRI) | 5’-CG*G AAT TC*A TGA TGC AGC ATA CTT CTG TGT-3’ |
| MCR-1TM-R | 5’-CTG GCT TAT GCA CCC GAA AGA AAC TGG CAT AAT GAC TGC-3’ |
| MCR-1OS-F | 5’-GCA GTC ATT ATG CCA GTT TCT TTC GGG TGC ATA AGC CAG-3’ |
| MCR-1-R (SalI) | 5’-CCG *GTC GAC* TCA GCG GAT GAA TGC GGT-3’ |
| MCR-2-F(EcoRI) | 5'-AACC *GAA TTC*ATGACA TCA CAT CAC TCT TG-3’ |
| MCR-2TM-R | 5’-GCA GCG GCT TAT GCA CGC GAA AGA AAC TCG CAT ACT GAC-3’ |
| MCR-2OS-F | 5’-GTC AGT ATG CGA GTT TCT TTC GCG TGC ATA AGC CGC TGC-3’ |
| CI-F | 5’- GCGAACACCAATCACTCATC-3’ |
| CI-R | 5’- CCAATCGGCACAAGCAACAG-3’ |
| MCR-2-R (SalI) | 5'-CCG *GTC GAC* TTA CTG GAT AAA TGC CGC GC-3’ |
| MCR-2-F(∆TM- EcoRI) | 5'-AAC CGA ATT CAT GAG TTT CTT TCG GGT GCA TAA G-3’ |
| MCR-2(E244A)-F | 5’-TTC GTC GTC GGT GCA ACG GCG CGT GCT-3’ |
| MCR-2(E244A)-R | 5’-AGC ACG CGC CGT TGC ACC GAC GAC GAA-3’ |
| MCR-2(T283A)-F | 5’-TGT GGC ACA TCG GCA GCG TAT TCT GTG-3’ |
| MCR-2(T283A)-R | 5’-CAC AGA ATA CGC TGC CGA TGT GCC ACA-3’ |
| MCR-2(H393A)-F | 5’-CAA ATG GGC AAT GCA GGG CCG GCG TAC-3’ |
| MCR-2(H393A)-R | 5’-GTA CGC CGG CCC TGC ATT GCC CAT TTG-3’ |
| MCR-2(D463A)-F | 5’-CTC TAT GTC AGT GCA CAC GGC GAG AGC-3’ |
| MCR-2(D463A)-R | 5’-GCT CTC GCC GTG TGC ACT GAC ATA GAG-3’ |
| MCR-2(H464A)-F | 5’-TAT GTC AGT GAC GCA GGC GAG AGC TTG-3’ |
| MCR-2(H464A)-R | 5’-CAA GCT CTC GCC TGC GTC ACT GAC ATA-3’ |
| MCR-2(H476A)-F | 5’-GGT GTC TAT CTG GCA GGT ATG CCA AAT-3’ |
| MCR-2(H476A)-R | 5’-ATT TGG CAT ACC TGC CAG ATA GAC ACC-3’ |

The restriction sites are letters underlined and italic. CI is an abbreviation for the circular intermediate.
