# Supplementary material for: Deciphering MCR-2 Colistin Resistance
Source: mBio. 2017 May 9;8(3):e00625-17. doi: 10.1128/mBio.00625-17 (PMC5424208; doi:10.1128/mBio.00625-17)
Supplement: TABLE S3 [file mbo003173304st3.docx]

**Table S3** Primers designed for *de novo* synthesis of the *mcr-2* gene

| **Primers** | **Sequences** |
| --- | --- |
| MCR-2-F  (EcoRI) | 5'-AACC *GAATTC* ATG ACA TCA CAT CAC TCT TGG-3’ |
| MCR-2-F1  (1-59) | 5’-ATG ACA TCA CAT CAC TCT TGG TAT CGC TAT TCT ATC AAT CCT TTT GTG CTG ATG GGT TT-3’ |
| MCR-2-F2 (41-99) | 5’-CTT TTG TGC TGA TGG GTT TGG TGG CGT TAT TTT TGG CAG CGA CAG CGA ACC TGA CAT TT-3’ |
| MCR-2-F3 (81-139) | 5’-GAC AGC GAA CCT GAC ATT TTT TGA AAA AGC GAT GGC GGT CTA TCC TGT ATC GGA TAA CT-3’ |
| MCR-2-F4 (121-179) | 5’-TAT CCT GTA TCG GAT AAC TTA GGC TTT ATC ATC TCA ATG GCG GTG GCG GTG ATG GGT GC -3’ |
| MCR-2-F5 (161-219) | 5’-CGG TGG CGG TGA TGG GTG CTA TGC TAC TGA TTG TCG TGC TGT TAT CCT ATC GCT ATG TG-3’ |
| MCR-2-F6 (201-259) | 5’-GTT ATC CTA TCG CTA TGT GCT AAA GCC TGT CCT GAT TTT GCT ACT GAT TAT GGG TGC GG-3’ |
| MCR-2-R6 (241-299) | 5’-TCA TAG ACC GTG CCA TAA GTA TCG GTA AAA TAG CTC GTC ACC GCA CCC ATA ATC AGT AG-3’ |
| MCR-2-F7 (281-339) | 5’-CTT ATG GCA CGG TCT ATG ACA CCA CCA TGC TCC AAA ATG CCA TGC AAA CCG ACC AAG CC-3’ |
| MCR-2-F8 (321-379) | 5’-CAT GCA AAC CGA CCA AGC CGA GTC TAA GGA CTT GAT GAA TTT GGC GTT TTT TGT GCG AA-3’ |
| MCR-2-F9 (361-419) | 5’-TTG GCG TTT TTT GTG CGA ATT ATC GGG CTT GGC GTG TTG CCA AGT GTG TTG GTC GCA GT-3’ |
| MCR-2-F10 (401-459) | 5’-CAA GTG TGT TGG TCG CAG TTG CCA AAG TCA ATT ATC CAA CAT GGG GCA AAG GTC TGA TT-3’ |
| MCR-2-F11 (441-499) | 5’-ATG GGG CAA AGG TCT GAT TCA GCG TGC GAT GAC ATG GGG TGT CAG CCT TGT GCT GTT GC-3’ |
| MCR-2-F12 (481-539) | 5’-GTC AGC CTT GTG CTG TTG CTT GTG CCG ATT GGA CTA TTT AGC AGT CAG TAT GCG AGT TT-3’ |
| MCR-2-R12 (521-579) | 5’-CGG GTT GAT ATA AAA ACG CAC TGG CTT ATG CAC CCG AAA GAA ACT CGC ATA CTG ACT GC-3’ |
| MCR-2-F13 (561-619) | 5’-GCG TTT TTA TAT CAA CCC GAT TAC GCC GAT TTA TTC GGT GGG TAA GCT TGC CAG TAT CG-3’ |
| MCR-2-F14 (601-659) | 5’-GGT AAG CTT GCC AGT ATC GAG TAC AAA AAA GCC ACT GCG CCA ACA GAC ACC ATC TAT CA-3’ |
| MCR-2-F15 (641-699) | 5’-CAA CAG ACA CCA TCT ATC ATG CCA AAG ACG CCG TGC AGA CCA CCA AGC CGA GCG AGC GT-3’ |
| MCR-2-F16 (681-739) | 5’-CAC CAA GCC GAG CGA GCG TAA GCC ACG CCT AGT GGT GTT CGT CGT CGG TGA GAC GGC GC-3’ |
| MCR-2-F17 (721-779) | 5’-GTC GTC GGT GAG ACG GCG CGT GCT GAC CAT GTG CAG TTC AAT GGC TAT GGC CGT GAG AC-3’ |
| MCR-2-F18 (761-819) | 5’-CAG AAT ACG CCG TCG ATG TGC CAC ACG ATG TCA CTT GGC TAA AAT TCG CCA AGC CAT CA-3’ |
| MCR-2-R18 (801-859) | 5’-CAG AAT ACG CCG TCG ATG TGC CAC ACG ATG TCA CTT GGC TAA AAT TCG CCA AGC CAT CA-3’ |
| MCR-2-F19 (841-899) | 5’-ACA TCG ACG GCG TAT TCT GTG CCG TGT ATG TTC AGC TAT TTG GGT CAA GAT GAC TAT GA-3’ |
| MCR-2-F20 (881-939) | 5’- TGG GTC AAG ATG ACT ATG ATGTCG ATA CCG CCA AAT ACC AAG AAA ATG TGC TAG ATA CG-3’ |
| MCR-2-F21 (921-979) | 5’-AGA AAA TGT GCT AGA TAC GCT TGA CCG CTT GGG TGT GGG TAT CTT GTG GCG TGA TAA TA-3’ |
| MCR-2-F22 (961-1019) | 5’- ATC TTG TGG CGT GAT AAT AAT TCA GAC TCA AAA GGC GTG ATG GAT AAG CTA CCT GCC AC-3’ |
| MCR-2-F23 (1001-1059) | 5’-TGG ATA AGC TAC CTG CCA CGC AGT ATT TTG ATT ATA AAT CAG CAA CCA ACA ATA CCA TC-3’ |
| MCR-2-F24 (1041-1099) | 5’-AGC AAC CAA CAA TAC CAT CTG TAA CAC CAA TCC CTA TAA CGA ATG CCG TGA TGT CGG TA-3’ |
| MCR-2-R24 (1081-1139) | 5’-CCA TTA TTG GCG CTG ACA TAG TCA TCT AGC CCG ACA AGC ATA CCG ACA TCA CGG CAT TC-3’ |
| MCR-2-F25 (1121-1179) | 5’-ATG TCA GCG CCA ATA ATG GCA AAG ATA TGC TCA TCA TGC TAC ACC AAA TGG GCA ATC AT-3’ |
| MCR-2-F26 (1161-1219) | 5’-ACA CCA AAT GGG CAA TCA TGG GCC GGC GTA CTT TAA GCG TTA TGA TGA GCA ATT TGC CA-3’ |
| MCR-2-F27 (1201-1259) | 5’-TAT GAT GAG CAA TTT GCC AAA TTC ACC CCC GTG TGC GAA GGC AAC GAG CTT GCC AAA TG-3’ |
| MCR-2-F28 (1241-1299) | 5’- GCA ACG AGC TTG CCA AAT GCG AAC ACC AAT CAC TCA TCA ATG CCT ATG ACA ATG CGC TA-3’ |
| MCR-2-F29 (1281-1339) | 5’- TGC CTA TGA CAA TGC GCT ACT TGC GAC TGA TGA TTT TAT CGC CAA AAG CAT CGA TTG GC-3’ |
| MCR-2-F30 (1321-1379) | 5’-GCC AAA AGC ATC GAT TGG CTA AAA ACG CAT GAA GCG AAC TAC GAT GTC GCC ATG CTC TA-3’ |
| MCR-2-R30 (1361-1419) | 5’-GAC ACC ATT TTC GCC CAA GCT CTC GCC GTG GTC ACT GAC ATA GAG CAT GGC GAC ATC GT-3’ |
| MCR-2-F31 (1401-1459) | 5’-CTT GGG CGA AAA TGG TGT CTA TCT GCA TGG TAT GCC AAA TGC CTT TGC ACC AAA AGA AC-3’ |
| MCR-2-F32 (1441-1499) | 5’-GCC TTT GCA CCA AAA GAA CAG CGA GCT GTG CCT GCG TTT TTT TGG TCA AAT AAT ACG AC-3’ |
| MCR-2-F33 (1481-1539) | 5’-TTT GGT CAA ATA ATA CGA CAT TCA AGC CAA CTG CCA GCG ATA CTG TGC TGA CGC ATG AT-3’ |
| MCR-2-F34 (1521-1579) | 5’-TAC TGT GCT GAC GCA TGA TGC GAT TAC GCC AAC ACT GCT TAA GCT GTT TGA TGT CAC AG-3’ |
| MCR-2-F35 (1561-1617) | 5’-AAG CTG TTT GAT GTC ACA GCG GGC AAG GTC AAA GAC CGC GCG GCA TTT ATC CAG TAA-3’ |
| MCR-2-R35 (1598-1617) | 5’-TTA CTG GAT AAA TGC CGC GC-3’ |
| MCR-2-R (SalI) | 5'-CCG *GTCGAC* TTA CTG GAT AAA TGC CGC GC-3’ |

*The underlined letters in italic denote restrictions sites.
