# Supplementary material for: Deciphering MCR-2 Colistin Resistance
Source: mBio. 2017 May 9;8(3):e00625-17. doi: 10.1128/mBio.00625-17 (PMC5424208; doi:10.1128/mBio.00625-17)
Supplement: TABLE S4 [file mbo003173304st4.docx]

**Table S4** Primers used for the *mcr-2* promoter synthesis

| **Primers** | **Sequences** |
| --- | --- |
| MCR-2(1)  (SalI) | 5'-ACGC GTCGAC TGC AAA CAA TCA TAA TCA CAT TAA CGG CAT TGA AAA CTT TTG GT-3’ |
| MCR-2(2) | 5’-CGG CAT TGA AAA CTT TTG GTC GCA ATC CAA ACG CAT TCT AAG AAA ATA TAA CGG TAT T-3’ |
| MCR-2(3) | 5’-TAA GAA AAT ATA ACG GTA TTG ACA AAA AGC ATT TTC ATT TAT TTA TCA AAG AAT GCG A-3’ |
| MCR-2(4) | 5’-TTA TTT ATC AAA GAA TGC GAG TTT AGA TTT AAC TAT GGC GCA CCA TCC AAC CAA TTA A-3’ |
| MCR-2(4)-R | 5’-TTA ATT GGT TGG ATG GTG CGC CAT AGT TAA ATC TAA ACT CGC ATT CTT TGA TAA ATA A-3’ |
| MCR-2(5) | 5’-CGC ACC ATC CAA CCA ATT AAG ATT ATT AAG AAA ATG GTG TGG GAT TTA AAG GTT TAT C-3’ |
| MCR-2(6) | 5’-GTG GGA TTT AAA GGT TTA TCT GGT ACA GCC CCT TTA TTT ATC TGC ACA ATA TAT CTA G-3’ |
| MCR-2(7) | 5’-TAT CTG CAC AAT ATA TCT AGA TTC TAC AAC GAC ATT GAA GTA TAA TCG CCA ACT TGT AT-3’ |
| MCR-2(8) | 5’-GTA TAA TCG CCA ACT TGT ATC GTA TAT GGC ATT TGT GGG TAA TTT CTA TG-3’ |
| MCR-2(8)-R  (EcoRI) | 5’-CCG *GAATTC* CAT AGA AAT TAC CCA CAA ATG CCA TAT ACG ATA CAA GTT GGC GAT TAT AC-3’ |
